# Reducing the Levels of Akt Activation by PDK1 Knock-in Mutation Protects Neuronal Cultures against Synthetic Amyloid-Beta Peptides

**DOI:** 10.3389/fnagi.2017.00435

**Published:** 2018-01-08

**Authors:** Shaobin Yang, Sònia Pascual-Guiral, Rebeca Ponce, Lydia Giménez-Llort, María A. Baltrons, Ottavio Arancio, Jose R. Palacio, Victoria M. Clos, Victor J. Yuste, Jose R. Bayascas

**Affiliations:** ^1^Unitat de Bioquímica de Medicina, Departament de Bioquímica i Biologia Molecular, Institut de Neurociències, Universitat Autònoma de Barcelona, Barcelona, Spain; ^2^Departament de Psiquiatria i Medicina Legal, Institut de Neurociències, Universitat Autònoma de Barcelona, Barcelona, Spain; ^3^Departament de Bioquímica i Biologia Molecular, Institute of Biotechnology and Biomedicine, Universitat Autònoma de Barcelona, Barcelona, Spain; ^4^Department of Pathology, The Taub Institute for Research on Alzheimer's Disease and the Aging Brain, Columbia University, New York, NY, United States; ^5^Departament de Biologia Cel.lular, Fisiologia i Immunologia, Institute of Biotechnology and Biomedicine, Universitat Autònoma de Barcelona, Barcelona, Spain; ^6^Departament de Farmacologia, Terapèutica i Toxicologia, Institut de Neurociències, Universitat Autònoma de Barcelona, Barcelona, Spain

**Keywords:** PDK1/Akt, knock-in mouse, Alzheimer disease, TACE α-secretase, unfolding protein response

## Abstract

The Akt kinase has been widely assumed for years as a key downstream effector of the PI3K signaling pathway in promoting neuronal survival. This notion was however challenged by the finding that neuronal survival responses were still preserved in mice with reduced Akt activity. Moreover, here we show that the Akt signaling is elevated in the aged brain of two different mice models of Alzheimer Disease. We manipulate the rate of Akt stimulation by employing knock-in mice expressing a mutant form of PDK1 (phosphoinositide-dependent protein kinase 1) with reduced, but not abolished, ability to activate Akt. We found increased membrane localization and activity of the TACE/ADAM17 α-secretase in the brain of the PDK1 mutant mice with concomitant TNFR1 processing, which provided neurons with resistance against TNFα-induced neurotoxicity. Opposite to the Alzheimer Disease transgenic mice, the PDK1 knock-in mice exhibited an age-dependent attenuation of the unfolding protein response, which protected the mutant neurons against endoplasmic reticulum stressors. Moreover, these two mechanisms cooperatively provide the mutant neurons with resistance against amyloid-beta oligomers, and might singularly also contribute to protect these mice against amyloid-beta pathology.

## Introduction

The PI3K (phosphoinositide 3-kinase) signaling pathway regulates cell and organism homeostasis by controlling fundamental cellular responses that include survival, differentiation, proliferation, growth, and metabolism (Engelman et al., 2006). Dysregulation of this signaling pathway leads invariably to pathological manifestations (Vanhaesebroeck et al., 2016). For example, increased PI3K activation is commonly found in tumor cells (Wong et al., 2010), whereas the inhibition of this signaling pathway compromise embryonic development and promotes diabetes in the adult life (Griffin et al., 2005; Foukas and Withers, 2010). Also, the activation of PI3K by insulin and IGF-1 is markedly disturbed in Alzheimer Disease (AD) brain, leading to sustained activation of Akt (Pei et al., 2003; Rickle et al., 2004), which in turns causes neurons to become increasingly and ultimately totally resistant to both agonists (Moloney et al., 2010; O'Neill et al., 2012; Talbot et al., 2012). This leads to inefficient GSK3β inhibition and Tau hyperphosphorylation (Ryder et al., 2004) promoting its self-aggregation (Lippens et al., 2007; Cowan et al., 2010).

Recently, a number of studies have evidenced that the modest attenuation of the PI3K signaling pathway might underlie physiological benefits in terms of cancer, metabolism regulation and aging (Bartke, 2008). For instance, mice overexpressing PTEN, the gene coding the phosphatase that antagonizes the pathway, were shown to be protected from cancer and aging (Ortega-Molina et al., 2012), whereas the increased lifespan and decrease age-related pathology associated to some life style strategies, such as dietary and caloric restriction, might rely on the limited activation of PI3K by insulin and/or IGF1 (Fontana et al., 2010). The activation of PI3K by BDNF plays a major role in the brain: BDNF mRNA and protein levels are decreased in samples of hippocampal and cortical tissues from AD patients compared with age-matched control subjects (Phillips et al., 1991). By contrast, increased serum levels of BDNF were also reported in both AD and Mild Cognitive Impairment (MCI) patients (Angelucci et al., 2009). These changes in the levels of BDNF may be well related to the different stages of AD pathology.

In fact, many extracellular molecules including growth factors, hormones and neurotransmitters signal through their specific receptors by activating PI3K, which in turn phosphorylates the membrane phospholipid phosphatydilinositol-(4,5)-bisphosphate (PtdIns(4,5)P_2_) at the carbon 3 within the intracellular inositol ring, to produce the phosphatydilinositol-(3,4,5)-trisphosphate (PtdIns(3,4,5)P_3_) second messenger (Vanhaesebroeck et al., 2001). The signal is terminated by PTEN, the phosphatase that breaks down the PtdIns(3,4,5)P_3_ to PtdIns(4,5)P_2_ (Leslie and Downes, 2004). Increases in the levels of PtdIns(3,4,5)P_3_ promote the activation of a number of effector serine-threonine protein kinases, all belonging to the AGC family. These include Akt, S6K, SGK, RSK, and PKC isoforms (Mora et al., 2004). PDK1 is the common master upstream kinase contributing to the activation of all these group of PI3K-stimulated AGC family members by phosphorylating their activation T-loops (Bayascas, 2010).

In the last decade, structural and genetical analysis allowed the rational design of knock-in mutation strategies to dissect the regulatory mechanisms of the PI3K/PDK1 downstream signaling pathway (Bayascas, 2008). Both Akt and PDK1 possess PH domains that bind to PtdIns(3,4,5)P_3_ and mediate in this manner the shuttling and co-localization of the two kinases at the plasma membrane, allowing PDK1 to activate Akt (Alessi et al., 1997). PtdIns(3,4,5)P_3_ promotes also the mTOR complexes-mediated phosphorylation of the different PI3K-stimulated AGC family members at a second activating site, the C-terminal hydrophobic motif, thereby creating a docking site for PDK1 binding, phosphorylation and activation (Biondi et al., 2002).

Mice expressing the mutant form of PDK1 K465E within the PH-domain incapable of PtdIns(3,4,5)P_3_ binding showed a defect in Akt activation (Bayascas et al., 2008), whereas in mice expressing the mutant form of PDK1 L155E within the pocket that recognizes the phosphorylated substrate docking site, PDK1 failed to activate most substrates with the exception of Akt (Bayascas et al., 2006). The PDK1^K465E/K465E^ mice were smaller but otherwise fertile and healthy, protected from diabetes (Bayascas et al., 2008), as well as protected from PTEN-induced tumorigenesis (Wullschleger et al., 2011). By contrast, the PDK1^L155E/L155E^ mice were embryonically lethal (Collins et al., 2003), whilst the restricted expression of the PDK1 L155E mutant protein to neuronal tissues resulted in microcephaly and abnormal cortical patterning, leading to cognitive deficits and disruptive behavior (Cordón-Barris et al., 2016).

Pietri and co-workers showed that the PDK1 kinase, the central switch in the PI3K signal transduction pathway, was hyperactivated in the brains of Alzheimer Disease (AD) patients, and that inhibition of PDK1 restored the cognitive defects and ameliorated the AD-like pathology in transgenic mice models of this disease (Pietri et al., 2013). The authors demonstrated that the inhibition of PDK1 resulted in the translocation of the Tumor necrosis factor-α (TNF-α)-converting enzyme (TACE) to the plasma membrane, which promoted TNFR1 and amyloid precursor protein (APP) shedding, thereby precluding TNFα toxicity and amyloid-β (Aβ) production. Although encouraging and promising, complete PDK1 inhibition had deleterious consequences, since mice started dying following 5 months of treatment (Pietri et al., 2013). Given the phenotypes of the PDK1 knock-in models, we postulate that inhibition of Akt entails beneficial consequences in terms of aging and thereby might have protected mice against amyloid-related pathology, whereas inhibition of the docking-site dependent PDK1 substrates might be responsible for the toxicity of the treatment.

To define the contribution of Akt to PDK1 actions along aging and in AD, a longitudinal study was conducted in which we genetically manipulated the levels of Akt activity by employing the PDK1^K465E/K465E^ knock-in mice with reduced Akt activation but otherwise intact PDK1 signaling. We find both the TACE protein levels and the TACE α-secretase catalytic activity increased in the membrane of the PDK1 mutant neurons and brain aged tissues, leading to increased TNFR1 shedding and reduced sensitivity to TNFα toxicity. We also provide evidence that, oppositely to the 3xTg-AD mice, the PERK/eIF2α axis within the unfolding protein response (UPR) is attenuated in the PDK1^K465E/K465E^ neurons and brain samples in an age-dependent manner, which confers cellular resistance against endoplasmic reticulum (ER) stressors such as tunicamycin. Moreover, the PDK1^K465E/K465E^ neurons are markedly protected against Aβ-induced neurotoxicity, and pharmacological inhibition of Akt protected wild type cells against Aβ-treatment. We propose a synergistic role of Akt for both the inhibition of TACE-mediated shedding of TNFR1 and the activation of the UPR in mediating Aβ toxicity, which highlights the potential of Akt rather than PDK1 inhibitors for AD treatment.

## Materials and methods

### Mice

The generation of the PDK1^K465E/K465E^ knock-in mice and the genotyping procedures were previously described (Bayascas et al., 2008). The 3xTg-AD mice colony was established by Dr. Giménez-Llort from progenitors kindly provided by Prof. Frank M. LaFerla (Oddo et al., 2003), whereas the brain samples from mice overexpressing APP (K670N:M671L) together with PS1 (M146L), termed APP/PS1 mice, line 6.2, were provided by Dr. Arancio at Columbia University (Holcomb et al., 1998). Transgenic male and female mice and matched non-transgenic wild type littermates were used in all the experiments. Mice were housed in pathogen-free facilities, 5–6 per cage, maintained on a 12-h light/dark cycle (lights on at 07:00) with *ad libitum* access to food and water. All animal procedures were performed in accordance with 2010/63/UE regarding the care and use of animals for experimental procedures following the recommendations of the Universitat Autònoma de Barcelona Ethical Committee for Human and Animal Experimentation. The protocols were approved by the Ethical Committee for Human and Animal Experimentation, Universitat Autònoma de Barcelona, and performed under a Generalitat de Catalunya Project License. The study complies with the ARRIVE guidelines developed by the NC3Rs (Kilkenny et al., 2010).

### Primary cultures of embryonic neurons and cell assays

Neuronal primary cultures were established from embryonic day 15,5 (E15,5) embryos as previously described (Zurashvili et al., 2013). Cortical cells were seeded at a density of 5 × 10^4^ cells/cm^2^ onto 50 μg/ml of poly-D-Lysine-coated 24-well plates for cell viability studies, or 6-well plates for western blot analysis, and grown for 6 days *in vitro* in a 5% CO_2_ incubator. The hippocampal cells were plated at a density of 2 × 10^4^ cells/cm^2^ on 24-well plates containing 12-mm glass coverslips coated with 150 μg/ml of poly-D-Lysine for 4 days *in vitro* in a 5% CO_2_ incubator. Stock solutions of tunicamycin and Akti-1/2 dissolved in dimethyl sulfoxide, or TNFα and Aβ dissolved in PBS, were added to cultures as indicated. Cell survival was determined by the MTT reduction assay as described previously (Zurashvili et al., 2013). For the quantification of apoptosis, cells were fixed in 2% paraformaldehyde, stained with 1 μg/ml of the DNA dye Hoechst 33342, and then visualized under the fluorescence microscope. Apoptosis was quantified at each condition point by scoring the percentage of cells exhibiting fragmented or condensed nuclei. At least 300 cells per well from 6 systematically collected fields starting from a random position were manually counted by two participants, with blinding to the treatment conditions.

### Generation of protein extracts and western blot analysis

Tissue extracts were prepared by homogenizing the frozen tissue in a 10-fold excess volume of ice-cold lysis buffer (50 mM Tris-HCl pH 7.5, 1 mM EGTA, 1 mM EDTA, 1 mM sodium orthovanadate, 50 mM sodium fluoride, 5 mM sodium pyrophosphate, 10 mM sodium-glycerophosphate, 0.27 M sucrose, 1% w/v Triton X-100, 0.1% v/v 2-mercaptoethanol, and a 1:100 dilution of protease inhibitor cocktail, (Sigma, Merck KGaA, Darmstadt, Germany) using the Polytron homogenizer; cultured cortical neurons were scrapped from wells in the same ice-cold lysis buffer. Lysates were centrifuged at 4°C for 15 min at 15,000 × g and supernatants aliquoted and preserved at −20°C. Protein concentrations were determined by the Bradford method using bovine serum albumin as standard. The activation state of the different pathways was assessed by loading 20 μg of protein extracts onto 6–10% polyacrylamide, 0.25M Tris-HCl pH 8.5, 0.1% (w/v) SDS gels casted in 10-well/1 mm-thick mini-PAGE (polyacrylamide gel electrophoresis) system (Bio-Rad, Hercules, California, USA), then electrophoresed on 25 mM Tris-HCl pH 8.5, 192 mM Glycine, 0.1% (w/v) SDS running buffer at 160 V for 60 min, and finally transferred to nitrocellulose membranes (Whatman, Maidstone, UK) on a 25 mM Tris-HCl pH 8.5, 192 mM Glycine, 20% methanol transfer buffer wet system at 100 V for 90 min. Samples were blocked with 10% skimmed milk, 1xTBS, 0.2% (w/v) Tween 20 for 1 h at room temperature, then immunoblotted over night at 4°C with 1 μg/ml of the indicated primary antibodies diluted in either 5% skimmed milk, 1xTBS, 0.2% (w/v) Tween 20 (total protein antibodies) or 0.5% BSA, 1xTBS, 0.2% (w/v) Tween 20 (phospho-antibodies), which were detected with a 1:5,000 dilution of the appropriate horseradish peroxidase-conjugated secondary antibodies diluted in 5% skimmed milk, 1xTBS, 0.2% (w/v) Tween 20. Membranes were incubated with the enhanced chemiluminescence (ECL) reagent, then either exposed to Super RX Fujifilm and developed, or detected using a ChemiDoc MP Imaging System (Bio-Rad, Hercules, California, USA), and quantified using the ImageJ software.

### Membrane and cytosol preparation

Cortex tissues were homogenized in 10 volumes of cold homogenization buffer (50 mM Tris-HCl pH 7.4, 0.27 M sucrose, 1 mM Na_3_VO_4_, 1 mM EDTA, 1 mM EGTA, 50 mM NaF, 5 mM sodium pyrophosphate, 10 mM sodium-glycerophosphate, and a 1:100 dilution of protease inhibitor cocktail (Sigma, Merck KGaA, Darmstadt, Germany) 50 strokes using a glass potter homogenizer. Homogenates were centrifuged at 1,000 × g for 15 min at 4°C to remove cellular debris. The cleared homogenates were centrifuged at 20,000 × g for 1 h at 4°C and the resultant supernatant collected as the cytosolic fraction; the pellet was resuspended and washed in the same volume of homogenization buffer at 20,000 × g for 1 h at 4°C. The supernatant was discarded and the pellet containing the membrane fraction resuspended in 1% SDS homogenization buffer.

### Preparation of amyloid-beta oligomers

Aβ oligomers were prepared as shown previously (Manterola et al., 2013). One mg of synthetic amyloid-β peptides (1-42) from amino acid 672–713 in the human amyloid precursor protein sequence (catalog number H-1368.1000, Bachem, Torrance, USA) was monomerized in hexafluor-2-propanol and then evaporated peptides resuspended in 20 μl of fresh anhydrous DMSO, vortexed 20 s, incubated 5 min at room temperature, and diluted with 424 μl of PBS to a final concentration of 500 μM. The solution was again vortexed, incubated at 4°C for 24 h, and then centrifuged at 14,000 × g 4°C for 10 min to remove insoluble pellets. The supernatant containing a mixture of soluble Aβ_1−42_ oligomers was collected and biochemically analyzed by Bicine/Tris/Urea SDS-PAGE (**Figure 9A**). 1.6M Tris-HCl pH 8.1, 8M urea, 0.4M H_2_SO_4_ resolving and 0.8M BisTris pH 6.7, 0.2M H_2_SO_4_ stacking gels were casted in 10-well/1 mm-thick mini PAGE system (Bio-Rad, Hercules, California, USA), then electrophoresed on 0.2M Bicine pH 8.2, 0.1M NaOH, 0.25% (w/v) SDS cathode and 0.2M Tris-HCl pH8.1, 0.05M H_2_SO_4_ anode running buffers at 100 V for 100 min, finally transferred to PVDF membranes (Whatman, Maidstone, UK) on a 10 mM CAPS pH 11, 10% methanol transfer buffer wet system at 200 mA for 90 min, and then immunoblotted as described.

### Tace immunoprecipitation and tace activity

1 μg of anti-TACE antibody was conjugated with 10 μl of Protein G Sepharose (GE Healthcare, Chicago, Illinois, USA) and incubated with 0.2 mg of pre-cleared tissue lysates overnight at 4°C on a shaking platform. The beads were washed three times with 1 ml of lysis buffer supplemented with 150 mM NaCl, resuspended in protein sample loading buffer, electrophoresed and then immunoblotted with the indicated antibodies. TACE activity was determined using the SensoLyte 520 TACE (α-Secretase) Activity Assay Kit (#AS-72085, AnaSpec, Inc., Fremont, USA) following the manufacturer's instructions. Reaction was started by adding 40 mM of the fluorophoric QXL520/5FAM FRET substrate and was allowed to proceed for 60 min. 30 μg of cortex and hippocampus, or 20 μg of cell lysate proteins were used. Fluorescence intensity was quantified in a fluorescence microplate reader (FLx800, BIO-TEK Instruments, Winooski, USA) at an excitation of 490 nm and emission of 520 nm. Results are expressed as the percentage of fold-change in fluorescence intensity compared to control samples.

### Quantification of BDNF and sTNFR1

Cortex and hippocampus BDNF levels were measured with the BDNF Quantikine ELISA Kit (#DBD00, R&D Systems Inc., Minneapolis, MN, USA) according to the manufacturer instructions. The levels of the cleaved soluble TNFR1 fragment were measured on either cortex and hippocampus protein extracts or cell culture medium by using the mouse sTNFR1 Quantikine ELISA Kit (#MRT10, R&D Systems, Inc., Minneapolis, USA) according to the manufacturer's instructions. Optical density of each well was measured using an automated microplate reader (GloMax-Multi Microplate Reader, Promega, Madison, Wisconsin, USA).

### Mouse cytokine/chemokine assays

Mouse Cytokine/Chemokine Magnetic Bead Panel (MCYTOMAG-70K-PMX, Millipore, Burlington, Massachusetts, USA) was employed according to manufacturer's instructions to assess the serum concentration of 24 different cytokines and chemokines. The data was obtained with the Xponent software and the cytokine/chemokine factor concentrations calculated based on the corresponding standard curves. All samples were analyzed in duplicate.

### Brain immunohistochemistry

Three 21-months old PDK1^+/+^ and PDK1^K465E/K465E^ matched mice were sacrificed by cervical dislocation and brains were immediately collected, frozen in isopentane cooled down to −80°C and stored at −80°C. Brains were embedded in O.C.T. Compound (Sakura Finetek, Tokyo, Japan) and sliced into 10 μm thick sagittal sections. Frozen sections were fixed in 4% paraformaldehyde for 40 min at 4°C. For antigen retrieval, sections were boiled 10 min in 10 mM sodium citrate pH 6, cooled down for 30 min on ice and then washed three times with Tris-buffered saline (TBS; 25 mM Tris pH 7.5, 150 mM NaCl). Samples were blocked with buffer containing 5% goat serum and 0.02% Triton in TBS for 30 min and incubated overnight at 4°C with primary antibodies diluted 1:100 in the same blocking buffer. Sections were washed three times with TBS buffer, incubated with the corresponding Alexa488 conjugated secondary antibodies diluted 1:400 in blocking buffer for 1.5 h at room temperature, and counterstained with 1 μg/ml Hoechst 33342 before mounting in Fluorsave reagent (Southern Biotec, Birmingham, USA). Immunostained sections were photographed with a Nikon Eclipse 90i epifluorescence microscope, the captured images were processed and the staining intensity was measured automatically along a grid line by using ImageJ 1.42q (Wayne Rasband, National Institutes of Health, USA) and Fiji (https://imagej.net/Fiji) softwares in 5 selected cortex or hippocampus sections per mouse using a sum projection of five Z-sections (1 μm/section), whereas Hoechst staining was used to determine the nuclear area for normalization.

### Immunocytochemistry

Dissociated cortical and hippocampal cells were fixed with 4% paraformaldehyde in PBS for 20 min at room temperature, washed three times with PBS for 5 min with slow agitation, permeabilized with 0.02% saponin in PBS for 7 min at room temperature, incubated 15 min in 0.01% saponin, 10 mM glycine in PBS, and then blocked in 0.01% saponin, 5% BSA, 10 mM glycine in PBS for 1 h. Primary antibodies were diluted 1:100 in PBS supplemented with 0.01% saponin-1% normal goat serum and incubated overnight at 4°C. Cells were then washed four times, corresponding secondary antibodies conjugated to Alexa488 fluorescent dye were used at a concentration of 1:400, and nuclei were stained with 1 μg/ml Hoechst 33342 for 90 min at room temperature. Cover slips were then mounted with FluoroSave Reagent on microscope slides to measure the intensity of the immunostaining signals as described in the immunohistochemistry section.

### Antibodies

The following antibodies were raised in sheep and affinity purified on the appropriate antigen. The Akt total antibody was raised against the sequence RPHFPQFSYSASGTA, corresponding to residues 466–480 of rat Akt; the total TSC2 antibody was raised against a sequence encompassing residues 1719–1814 of mouse TSC2; the total PRAS40 antibody was raised against the peptide DLPRPRLNTSDFQKLKRKY, corresponding to residues 238–256 of human PRAS40; total TrkB (catalog number 4603), total ERK (catalog number 9102), phospho-Akt Thr308 (catalog number 9275), phospho-Akt Ser473 (catalog number 9271), phospho-GSK3α/β Ser21/9 (catalog number 9331), phospho-FOXO1 Ser256 (catalog number 9461), total FOXO1 (catalog number 2880), phospho-TSC2 Thr1462 (catalog number 3611), phospho-PRAS40 Thr246 (catalog number 2997), phospho-PDK1 Ser241 (catalog number 3061), total PDK1 (catalog number 3062), phospho-Threonine (catalog number 9381), phospho-PERK Thr980 (catalog number 3179), total PERK (catalog number 5683), phospho-eIF2α Ser51 (catalog number 3597), BiP (catalog number 3177), IRE1α (catalog number 3294), CHOP (catalog number 2895) and PDI (catalog number 3501) antibodies were purchased from Cell Signaling Technology, Danvers, Massachusetts, USA. Total GSK3α/β (catalog number sc-7291) antibody was purchased from Santa Cruz Biotechnology, Dallas, Texas, USA. TACE antibody was purchased from QED Bioscience, San Diego, California, USA (catalog number QEB-1131). TNFR1 antibody was obtained from MBL International, Woburn, Massachusetts, USA (catalog number JM-3125). GAPDH antibody was from Abcam, Cambridge, UK (catalog number ab8245). Aβ (6E10) monoclonal antibody was obtain from Biolegend, San Diego, California, USA (catalog number Sig-39345-200). APP antibody was purchased from Sigma, Merck KGaA, Darmstadt, Germany (catalog number 8717). For immunoblot analysis, appropriate secondary antibodies coupled to horseradish peroxidase were obtained from Thermo, Waltham, Massachusetts, USA. For immunohistochemistry and immunocytochemistry analysis, the Alexa Fluor 488-conjugated goat anti-rabbit secondary antibody was obtained from Invitrogen, Waltham, Massachusetts, USA (catalog number A11008).

### Statistical analysis

Data was analyzed using GraphPad software (GraphPad Software Inc., La Jolla, CA, USA). Statistical significance of the difference between two groups was determined by using the unpaired, two tailed Student's *t*-test analysis. Multiple groups were assessed by One-way analysis of variance (ANOVA), and the statistical significance of the differences between categories analyzed by *Post-hoc* using Tukey test. Results are presented as the mean ± standard error of the mean. *P*-values of < 0.05 and < 0.005 between categories were considered significant and are indicated in the figures.

## Results

### Hyperactivation of the PDK1/Akt signaling axis in AD transgenic mice models

**Figure 1:**
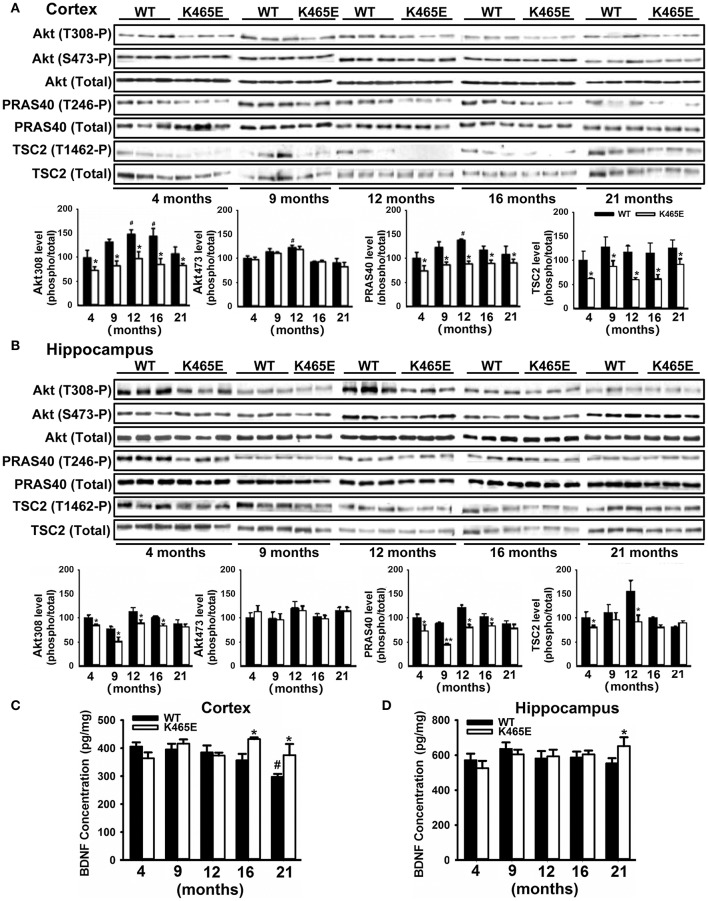
Reduced Akt activity in the PDK1^K465E/K465E^ mice brain. Cortical **(A)** and hippocampal **(B)** tissues from PDK1^+/+^ wild type (WT, black bars) and PDK1^K465E/K465E^ mutant (K465E, white bars) mice of the depicted age in months were subjected to immunoblot analysis with the indicated phospho- and total antibodies. Each lane corresponds to a sample derived from a different mouse. Band densitometry quantification of the ratio between phosphorylated and total protein levels is shown at the bottom of each panel, where values are represented as percentage of 4-months old wild type controls. The concentration of BDNF was measured in the cortex **(C)** and hippocampus **(D)** from the same PDK1^+/+^ wild type (WT) and PDK1^K465E/K465E^ mutant (K465E) mice at the indicated age in months. The data are represented as the mean ± SEM for at least three different mice per genotype. ^*^p < 0.05 (Student's *t*-test) between mutants and controls. ^#^*p* < 0.05 (Tukey test) compared to 4 months of age.

AD is an age-related neurodegenerative disorder disturbing the function of particular brain regions such as cortex and hippocampus (Huang and Mucke, 2012). To investigate the importance of the PDK1/Akt signaling in AD, we took advantage of the PDK1^K465E/K465E^ mice, in which we showed that BDNF-mediated activation of Akt was impaired in a time and dose-dependent manner (Zurashvili et al., 2013). As expected, mutation of the PDK1 phosphoinositide binding site in the PDK1^K465E/K465E^ mice caused a reduction in the levels of Akt activity along the whole adulthood both in the cortex (Figure 1A) and the hippocampus (Figure 1B). This is denoted by the decrease in the levels of phosphorylation of the Akt substrates PRAS40 at the Thr246 and TSC2 at the Thr1462 specific Akt sites, which originated from the decreased phosphorylation of Akt at the Thr308 residue within the activation loop by PDK1, but not the Ser473 within the hydrophobic motif domain that is phosphorylated by mTORC2. Of note, the deficits in Akt signaling tend to be attenuated in the aged 16–21 months-old mice brain, which correlated with elevated levels of BDNF both in the cortex (Figure 1C) and the hippocampus (Figure 1D) mutant tissue extracts.

**Figure 2:**
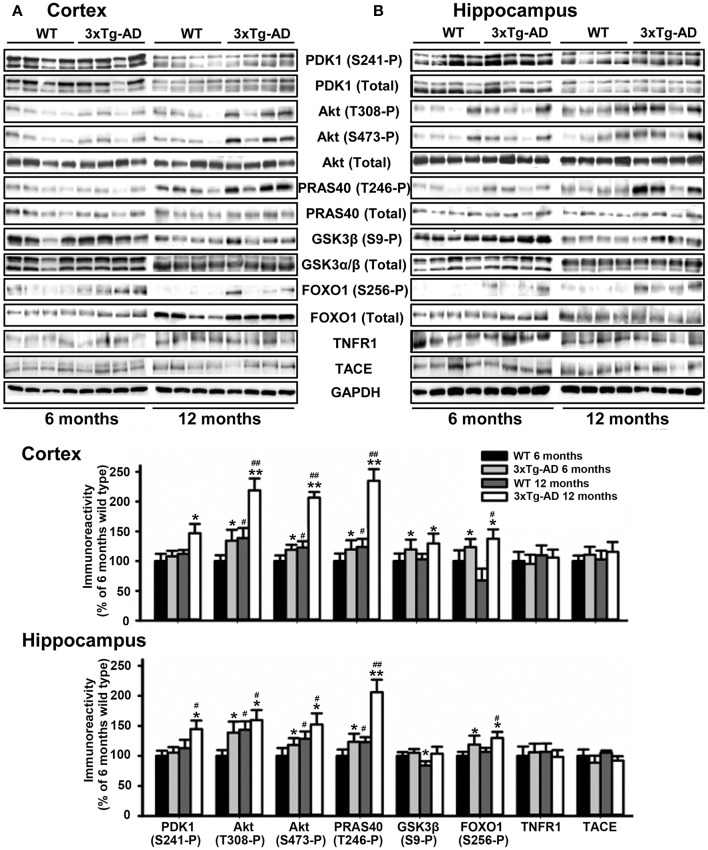
Hyperactivation of the Akt signaling in the 3xTg-AD mice brain. Cortex **(A)** and hippocampus **(B)** protein extracts obtained from wild type (WT) and triple transgenic AD mice model (3xTg-AD) at 6 and 12 months of age were subjected to immunoblot analysis with the indicated phospho and total antibodies. Each lane corresponds to a sample derived from a different mouse. Band densitometry quantification of the ratio between phosphorylated and total protein levels represented as percentage of 6-months old wild type controls is shown below the panels. The data are represented as the mean ± SEM for at least three different mice per genotype. ^*^p < 0.05 and ^**^p < 0.005 compared to controls as obtained by the Student's *t*-test. ^#^*p* < 0.05 and ^##^*p* < 0.005 (Tukey test) compared to 6 months of age.

We next monitored the degree of activation of this signaling pathway in brain tissues derived from 3xTg-AD and APP/PS1 transgenic mice and their corresponding matched control littermates at different ages of life. Strikingly, the levels of autophosphorylation of PDK1 at the Ser241 activation loop site, and those of Akt at both Thr308 and Ser473 activating residues were 50% higher both in the cortex (Figure 2A) and the hippocampus (Figure 2B) of the 3xTg-AD transgenic mice, which was accompanied by similar increase in the phosphorylation of the Akt substrate PRAS40 at Thr246 and, to a lesser extent, of GSK3β at Ser9 and FOXO1 at Ser256, their specific Akt sites. This biochemical alteration was already detectable in the 3xTg-AD transgenic mice at pre-pathological stages of the disease (6 months of age) and became accentuated by 12 months of age when the symptoms are overt (Oddo et al., 2008). By contrast, the PDK1/Akt signaling was found slightly attenuated in the cortex of the APP/PS1 mice at 6 months of age (Supplementary Figure 1A), and up to 2-fold increased by 12 months of age (Supplementary Figure 1B).

### Increased tace activity and elevated soluble TNFR1 levels in the aged PDK1^K465E/K465E^ knock-in mice cortex and hippocampus

Aging is the most prominent non-genetic risk factor for AD and is characterized by a progressive deterioration in the homeostasis of the neuro-immuno-endocrine systems. In particular, the increase in the secretion of inflammatory cytokines may play a vital role in the occurrence and development of AD (Wang et al., 2015). We therefore profiled the serum levels of a number of cytokines in the PDK1^K465E/K465E^ mice compared to PDK1^+/+^ control littermate mice at different ages of life. Interestingly, the levels of IL-2, IL-9, and TNFα, three cytokines commonly elevated in AD, were also 10-, 4- and 2-fold increased respectively in the PDK1^+/+^ control aged mice, but not in the PDK1^K465E/K465E^ mutant mice (Supplementary Figure 2).

**Figure 3:**
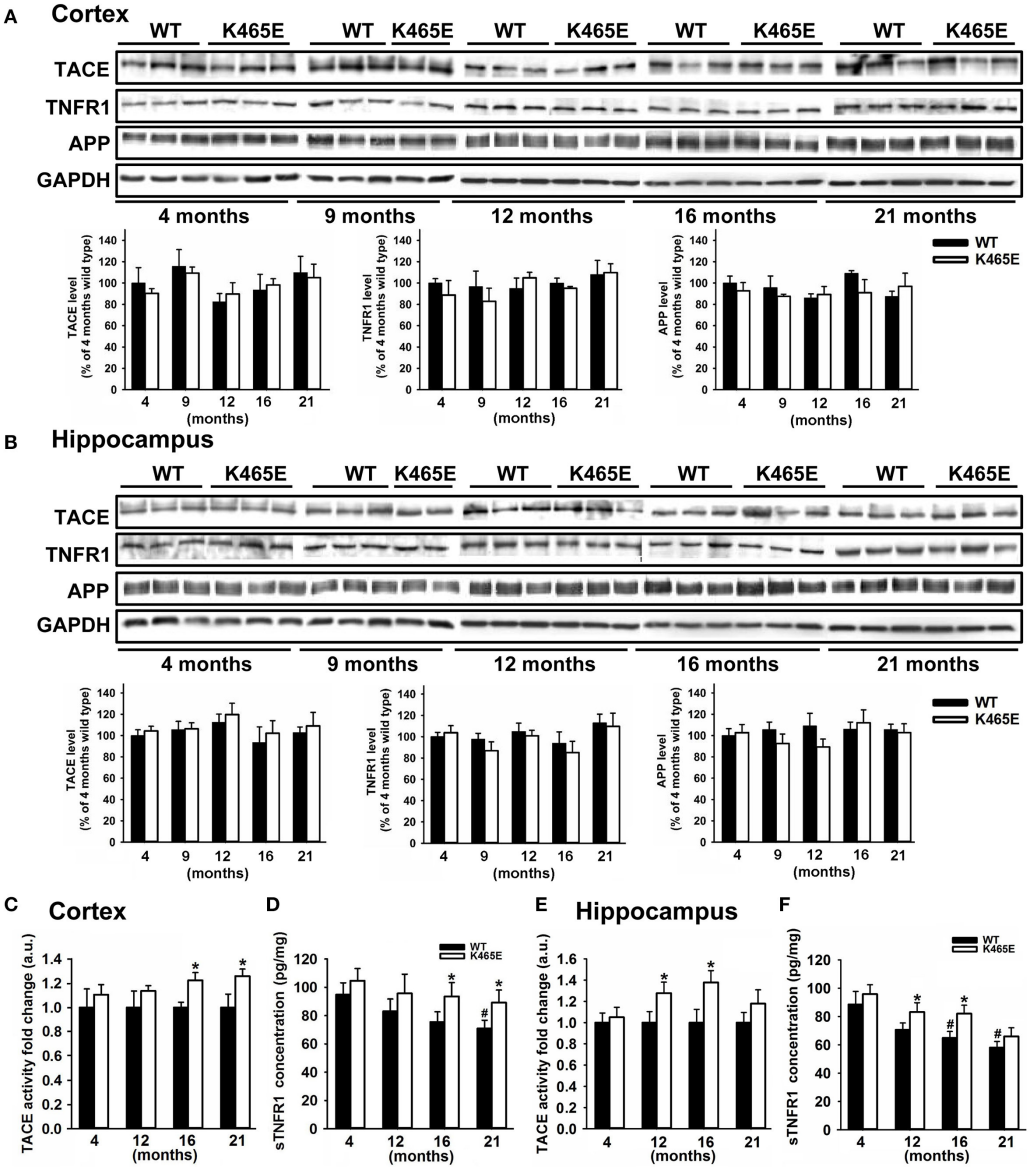
Increased TACE α-secretase activity and soluble TNFR1 levels in the aged PDK1^K465E/K465E^ mice cortex. Cortical **(A)** and hippocampal **(B)** tissue extracts from PDK1^+/+^ wild type (WT, black bars) and PDK1^K465E/K465E^ mutant (K465E, white bars) mice of the indicated age in months were subjected to immunoblot analysis with the described antibodies. Each lane corresponds to a sample derived from a different mouse. Band densitometry quantification normalized to GAPDH levels is shown at the bottom and expressed as a percentage of the 4 months-old wild type controls. The TACE α-secretase activity **(C,E)** and concentration of soluble TNFR1 **(D,F)** were measured on cortical and hippocampal protein extracts from the same PDK1^+/+^ wild type (WT) and PDK1^K465E/K465E^ mutant (K465E) mice at the indicated age in months. The data are represented as the mean ± SEM for at least three different mice per genotype. ^*^p < 0.05 compared with wild types as obtained by the Student's *t*-test. ^#^*p* < 0.05 compared to 4 months of age as obtained by the Tukey test.

We focused our attention on TNFα, an inflammatory cytokine acting through two specific transmembrane receptors, termed TNFR1 and 2, which have been reportedly shown to contribute to AD-related brain inflammation and to modulate neuronal viability (Yang et al., 2002; Li et al., 2004). TACE mediates the shedding of TNF-α and the two TNF-α receptors TNFR1 and TNFR2 (Gooz, 2010), as well as the α-secretase-mediated cleavage of APP (Buxbaum et al., 1998). We first analyzed by western blot the levels of expression of TACE, TNFR1 and APP in cortical (Figure 3A) and hippocampal (Figure 3B) total protein extracts derived from PDK1^K465E/K465E^ mutant and PDK1^+/+^ control littermate mice at different ages of life, and found no differences between genotypes along the entire adulthood. Likewise, unaltered TACE and TNFR1 protein levels were also observed in the cortex and the hippocampus of 3xTg-AD (Figure 2) and in the cortex of the APP/PS1 (Supplementary Figure 1) transgenic mice at either 6 or 12 months of age.

We next determined the TACE α-secretase catalytic activity levels in cortical and hippocampal protein extracts, which were around 20% higher in the cortex of the 16- and 21-month old and at 12- and 16-month in the hippocampus of the aged PDK1^K465E/K465E^ mice compared to the young mutant mice samples or the PDK1^+/+^ control littermate mice (Figures 3C,E). In agreement with that, the soluble TNFR1 levels were accordingly increased both in the cortex and the hippocampus of the PDK1^K465E/K465E^ knock-in mice compared to the controls (Figures 3D,F).

### Increased levels of tace with decreased TNFR1 levels in the plasma membrane of the PDK1^K465E/K465E^ knock-in mice neurons

**Figure 4:**
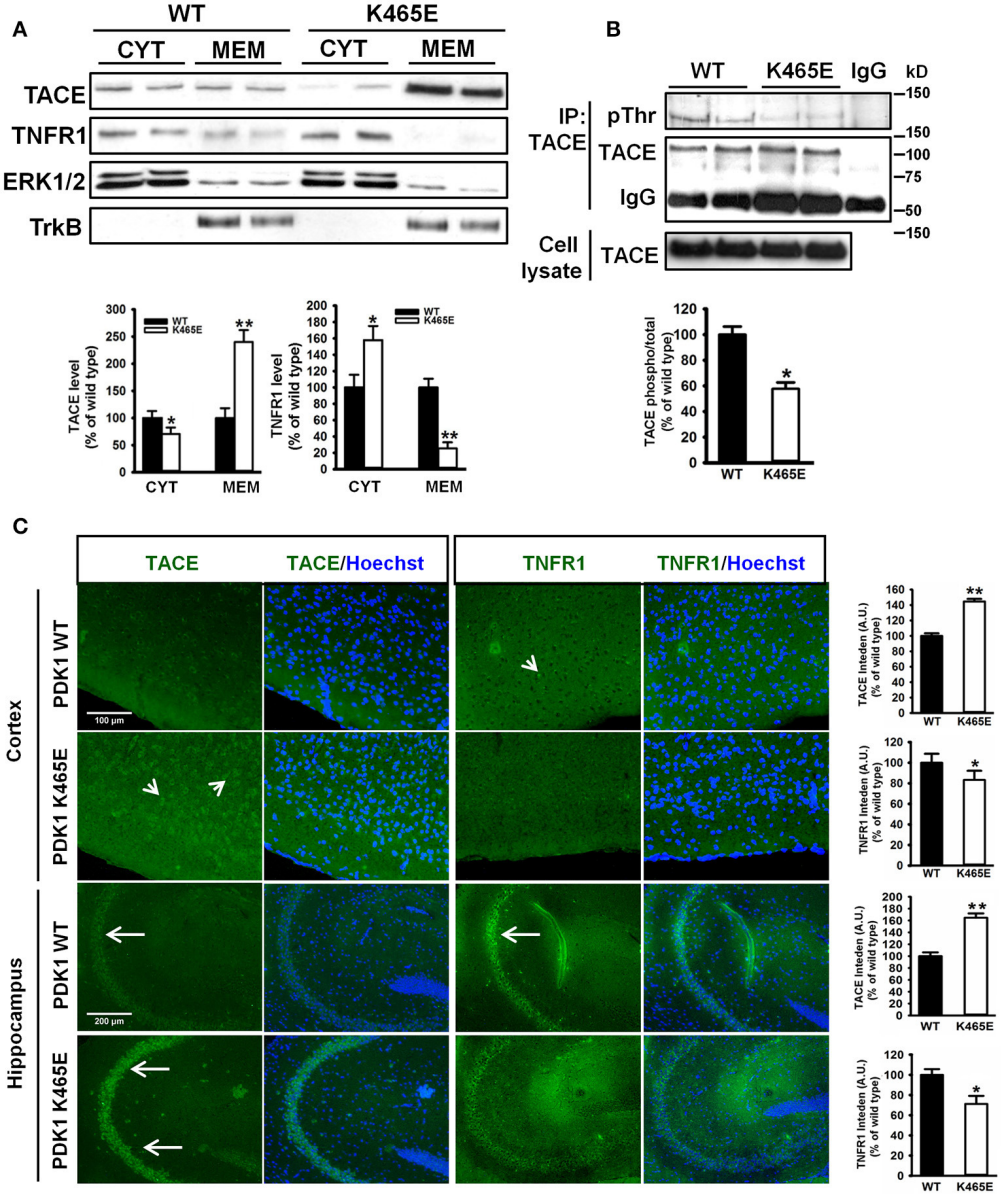
Increased TACE and decreased TNFR1 protein levels at the plasma membrane in the PDK1^K465E/K465E^ knock-in mice brain. **(A)** Brain cytosolic (CYT) and membrane (MEM) subcellular fractions obtained from PDK1^+/+^ wild type (WT, black bars) and PDK1^K465E/K465E^ mutant (K465E, white bars) 21-months old mice were immunoblotted with the indicated antibodies, where ERK1/2 and TrkB are employed as cytosolic and membrane markers, respectively. **(B)** TACE was immunoprecipitated (IP TACE) with specific antibodies from total brain protein extracts from PDK1^+/+^ wild type (WT) and PDK1^K465E/K465E^ mutant (K465E) 21-months old mice and immunoblotted both with TACE and pan-pThr antibodies, as depicted. The levels of TACE in the total cellular lysates and the immunoglobulin controls (IgG) are also shown. **(A,B)** Representative Western blots of three independent experiments are shown, and the band densitometry quantification expressed as a percentage of the wild type controls is plotted at the bottom. **(C)** Representative epifluorescence micrographs of cortical and CA3 middle hippocampal sections obtained from PDK1^+/+^ wild type (WT) and PDK1^K465E/K465E^ mutant (K465E) 21-months old mice brain stained with TACE, TNFR1, and the nuclear Hoechst dye; arrows indicate the specific surface staining; the intensity of the signals were quantified and expressed as a percentage of the wild type controls. The data are represented as the mean ± SEM for at least three different mice per genotype. ^*^p < 0.05 and ^**^p < 0.005 compared with wild types as obtained by the Student's *t*-test.

The α-secretase activity of TACE is mainly regulated by controlling its localization to the plasma membrane (Edwards et al., 2009). Phosphorylation of TACE at the C-terminus cytosolic domain by different upstream kinases modulate TACE trafficking (Gooz, 2010). Among them, PDK1 has been proposed to promote TACE phosphorylation and internalization (Pietri et al., 2013). Given the increased cortical TACE α-secretase activity observed in the PDK1^K465E/K465E^ mutant samples in which the Akt kinase activity is reduced, we sought to determine the consequences of this signaling lesion for TACE subcellular localization. TACE protein levels were three times more abundant in the membrane fractions of the PDK1^K465E/K465E^ 21-months old mice brain when compared to their corresponding controls, which was accompanied by a nearly absence of TNFR1 protein in those subcellular fractions (Figure 4A). This was corroborated by immunohistochemical analysis, which revealed a 40% increase in TACE surface staining and a 20% decreased detection of TNFR1 in the 21-months old PDK1^K465E/K465E^ mice brain cortical and hippocampal sections compared to the controls (Figure 4C). Of note, the levels of phosphorylation of TACE, as detected with antibodies raised against phosphorylated threonine residues, were nearly 2-fold reduced in the PDK1^K465E/K465E^ TACE immunoprecipitates compared to the controls, thereby raising the possibility of Akt being a major upstream kinase downstream of PDK1 governing the phosphorylation and shuttling of TACE (Figure 4B).

### Reduced vulnerability of the PDK1^K465E/K465E^ primary cortical neurons to TNFα–induced toxicity

**Figure 5:**
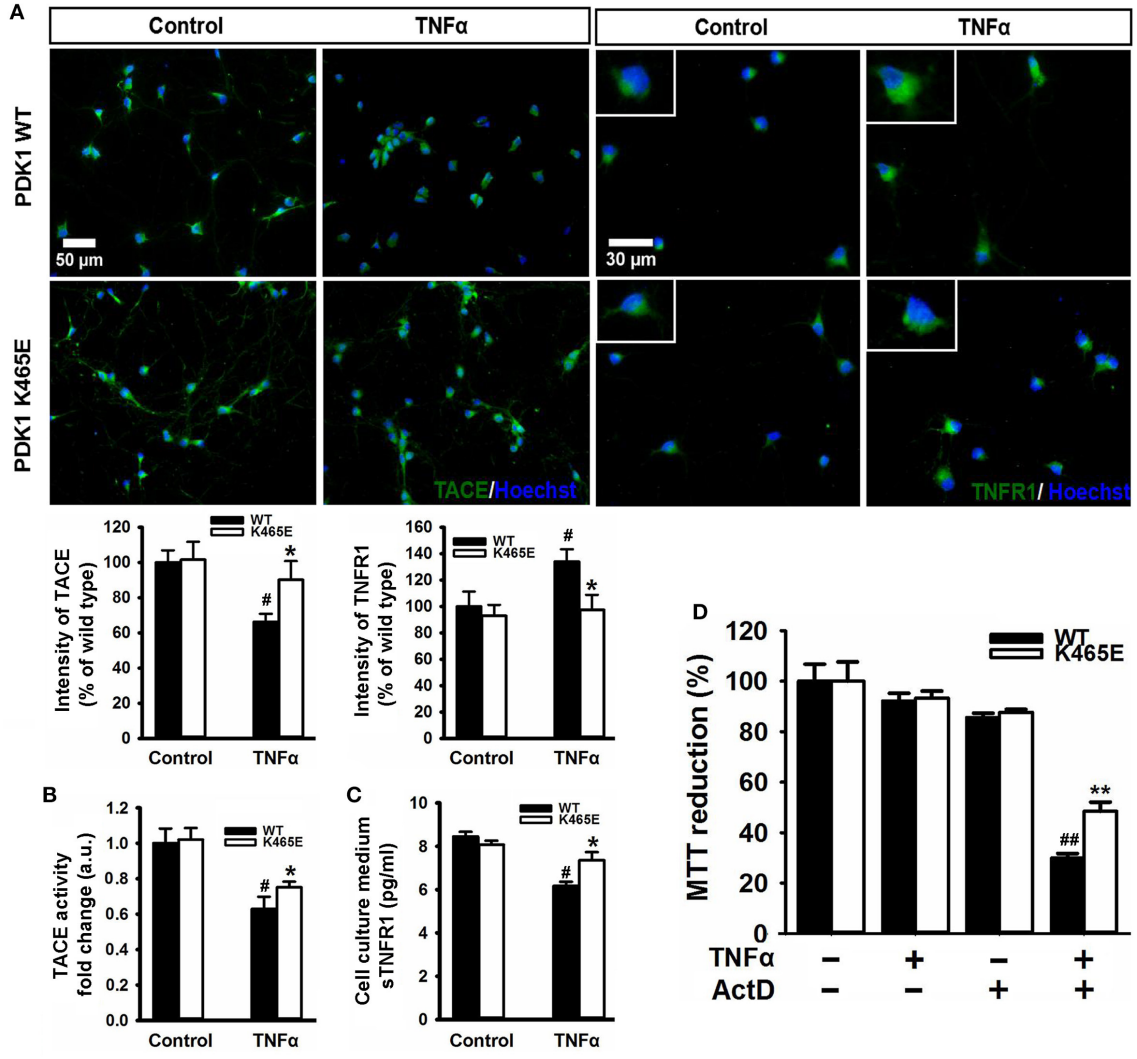
Reduced TNFα–induced cell death in the PDK1^K465E/K465E^ neurons. Primary cultures of cortical neurons obtained from PDK1^+/+^ wild type (WT, black bars) and PDK1^K465E/K465E^ mutant (K465E, white bars) E15,5 embryos were grown for 6 days *in vitro* and then treated or not with 100 ng/ml of recombinant TNFα and/or1 nM Actinomycin D for 24 h, as indicated. **(A)** Representative epifluorescence images stained with TACE or TNFR1 and merged with the nuclear Hoechst dye, as indicated; bars, 50 and 30 μm for TACE and TNFR1, respectively; quantification of the cell surface signals is shown below as the mean ± SEM for 200 neurons from four different embryos per condition, and expressed as a percentage of the wild type controls. The TACE α-secretase activity **(B)** and the levels of soluble TNFR1 in the culture medium **(C)** were also determined and expressed as the mean ± SEM from four different embryos per condition. **(D)** Cell viability was determined by the MTT reduction assay and represented as the mean ± SEM for at least five independent embryos per genotype from two separate experiments, with each sample assayed in triplicate. ^*^p < 0.05 and ^**^p < 0.005 compared with wild types as obtained by the Student's *t*-test. ^#^*p* < 0.05 and ^##^*p* < 0.005 between controls and treatments analyzed by the Tukey test.

TNFα is synthesized as a membrane-bound pro-protein which is processed by TACE to generate the mature cytokine. Since TNFα can elicit pro-apoptotic signals acting through TNFR1, we next aimed to determine whether the increased TNFR1 shedding observed in the PDK1 mutant mice brain could be instrumental in protecting primary cultures of cortical neurons against TNFα toxicity. Immunocytochemical analysis of cortical neurons exposed to 100 ng/ml TNFα for 24 h revealed a marked 40% decrease in the levels of TACE and a clear 40% increase in the levels of membrane-bound TNFR1 in the wild type samples, but not in the mutant cultures (Figure 5A). Accordingly, similar reduction both in the α-secretase activity of TACE as well as in the concentration of soluble TNFR1 released to the culture media was observed in the wild type cells, which was significantly attenuated in the mutant neurons (Figures 5B,C). In spite of these data, TNFα failed to compromise cell viability both in the wild type and the mutant cultures (Figure 5D). Neurons can overcome the pro-apoptotic signals dictated by TNFα by expressing the anti-apoptotic Bcl-2 family member Bcl-XL (Gozzelino et al., 2008). Treatment of the cells with TNFα in the presence of the broad RNA synthesis inhibitor Actinomycin-D drastically reduced the cell viability of the wild type cultures, as denoted by a 70% decrease in the MTT assay values, but to a lesser extent in the mutant cells, which exhibited a significant 50% decrease instead.

### Age-dependent attenuation of the PERK/EIF2A axis within the unfolding protein response in the PDK1^K465E/K465E^ mice

**Figure 6:**
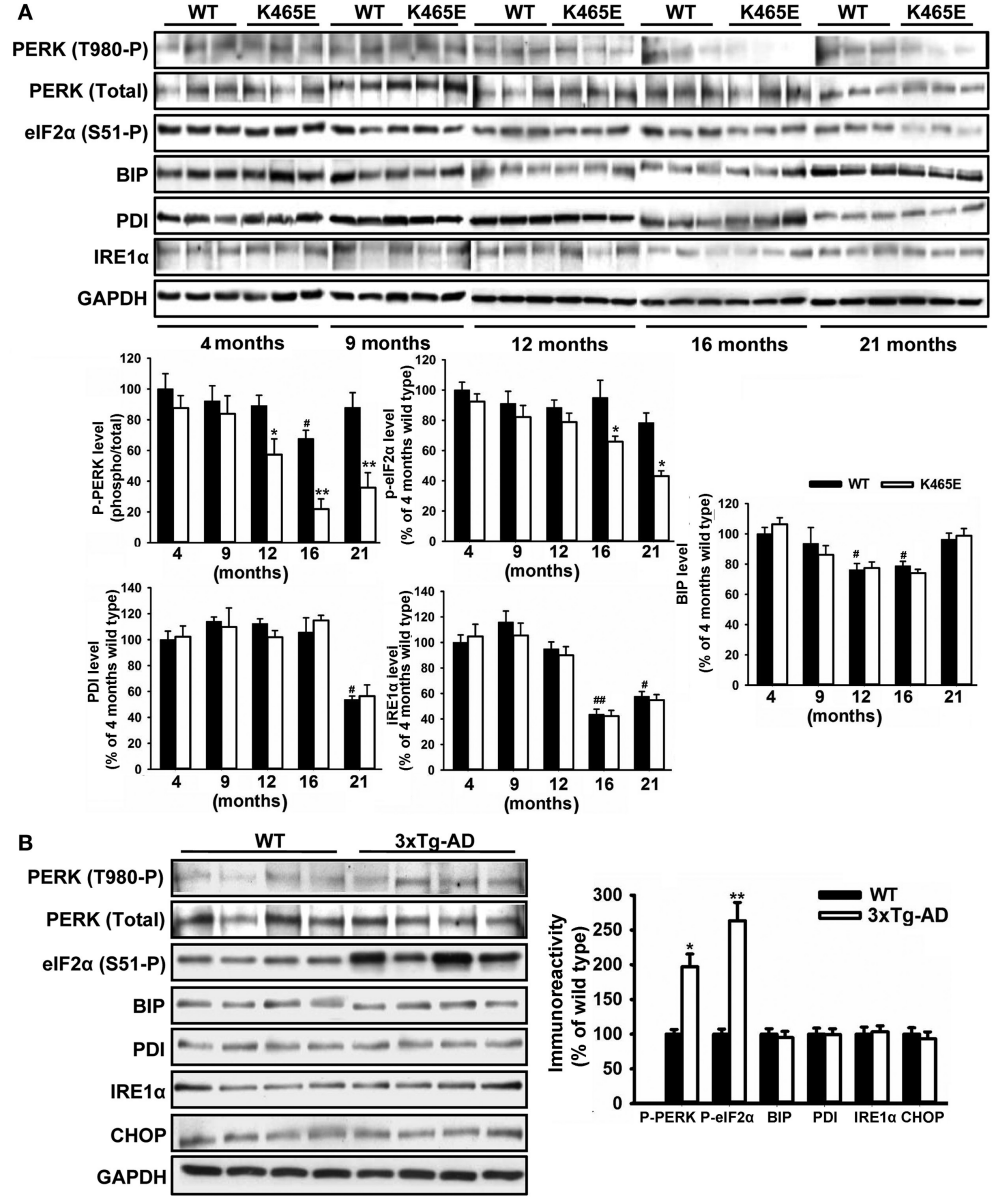
Opposite changes of the PERK/eIF2α axis within the UPR in the PDK1^K465E/K465E^ mice and the 3xTg-AD mice. Cortical brain extracts from PDK1^+/+^ wild type (WT, black bars) and PDK1^K465E/K465E^ mutant (K465E, white bars) mice of the different ages **(A)**, or 12-months old wild type (WT) and triple transgenic AD mice (3xTg-AD) model **(B)**, were subjected to immunoblot analysis with the indicated antibodies. Each lane corresponds to a sample derived from a different mouse. Band densitometry quantification of the ratio between the indicated markers and control levels is shown at the bottom **(A)** or at the right **(B)** expressed as a percentage of the 4-months old wild type controls. ^*^p < 0.05 or ^**^p < 0.005 compared with wild types as obtained by the Student's *t*-test. ^#^*p* < 0.05 and ^##^*p* < 0.005 compared to 4 months of age as obtained by the Tukey test.

The accumulation of misfolded proteins is a common characteristic of neurodegenerative syndromes such as AD, and alterations in the ability of the endoplasmic reticulum (ER) to counteract this stress through the unfolding protein response (UPR) could represent a molecular connection among these neurodegenerative diseases (Mercado et al., 2013). This adaptive response consists on a complex signaling network restoring homeostasis or triggering apoptotic cell dead depending on the intensity of the insult. We monitored the UPR by western blot analysis with specific antibodies against different elements on this signaling cascade. Among them, we consistently found up to 5-fold decreased levels of phosphorylation of the PERK kinase at Thr980 within the activation segment, which was accompanied by a 2-fold reduction in the levels of phosphorylation of the eIF2α substrate at the specific Ser51 PERK site, in the cortex of the PDK1^K465E/K465E^ aged mice compared to either the wild type or the young mice (Figure 6A). In sharp contrast, the levels of phosphorylation of both PERK at Thr980 and eIF2α at Ser51 were increased by 2-fold in the cortex of the 12-months old 3xTg-AD mice (Figure 6B), whereas the protein levels of other components of the UPR analyzed such as BIP, PDI, IRE1α or CHOP remained unchanged between genotypes.

### Attenuated UPR signaling in the PDK1^K465E/K465E^ primary cortical neurons confers resistance to ER stress

**Figure 7:**
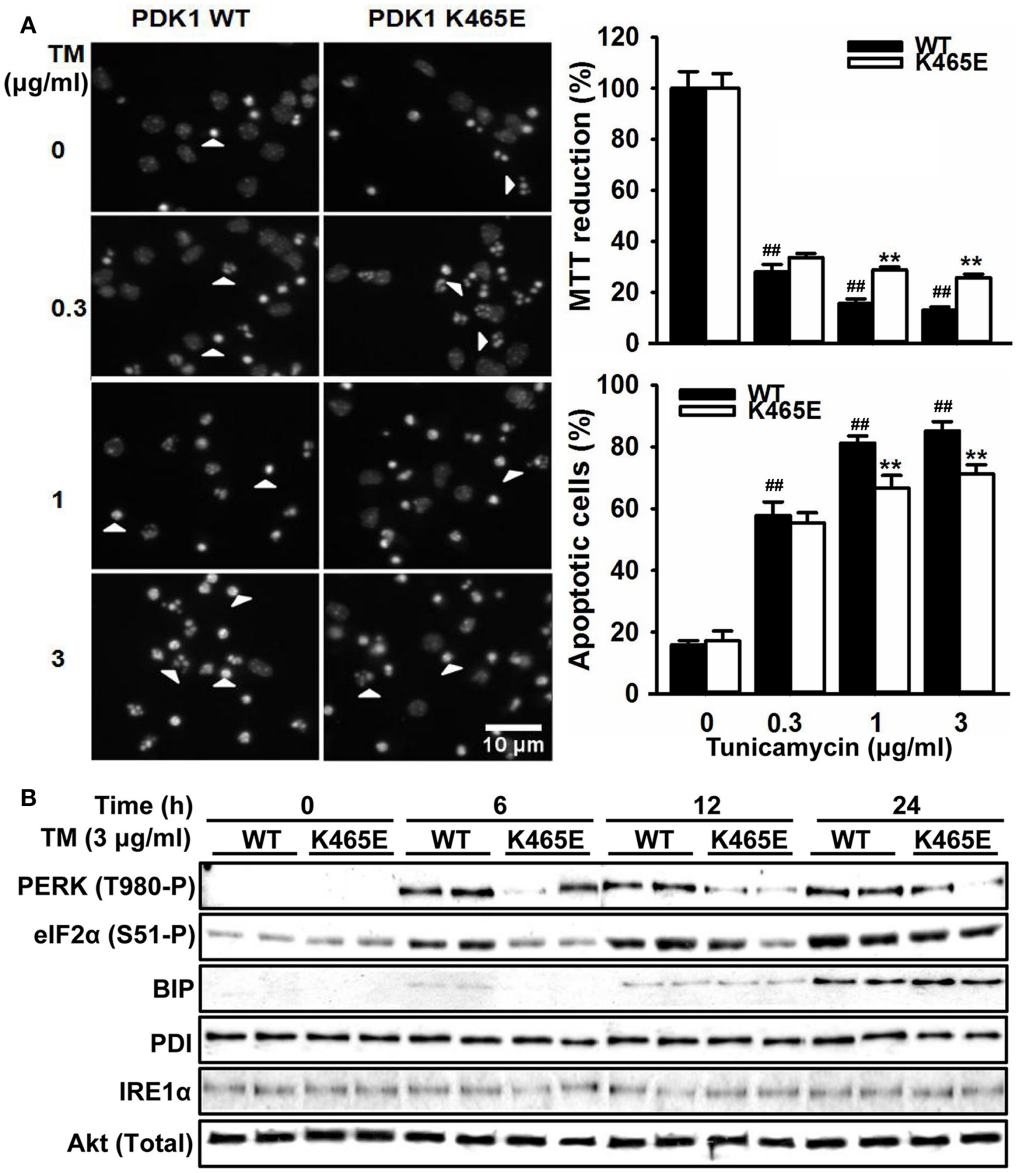
Attenuated UPR signaling protects PDK1^K465E/K465E^ neurons against tunicamycin-induced ER stress. Primary cultures of cortical neurons obtained from PDK1^+/+^ wild type (WT, black bars) and PDK1^K465E/K465E^ mutant (K465E, white bars) E15,5 embryos were grown for 6 days *in vitro* and then treated with the indicated doses of tunicamycin (TM) for 24 h **(A)**, or with 3 μg/ml of tunicamycin for the indicated time points **(B)**. **(A)** left panel, representative micrographs of Hoechst-stained cortical cultures, where arrowheads indicate apoptotic nuclei, scale bar 10 μm; right upper panel, the cell viability was determined with the MTT reduction assay and is expressed as a percentage of the untreated cells; right bottom panel, the percentage of apoptotic cells was obtained by scoring the number of nuclei exhibiting chromatin fragmentation divided by the total. Data represents the mean ± SEM for at least five independent embryos per genotype from two separate experiments, with each sample assayed in triplicate. ^**^p < 0.005 compared with the corresponding wild types for each condition as obtained by the Student's *t*-test. ^##^*p* < 0.005 (Tukey test) statistically significant differences between controls and treatments. **(B)** Protein extracts were obtained from the indicated cortical cells and then immunoblotted with the depicted antibodies, where total Akt was employed as a loading control.

The hyperactivation of the PERK/eIF2α signaling observed in the 3xTg-AD mice cortex is most likely caused by the exposure of the neurons to elevated levels of Aβ peptides, which could contribute to the neuronal loss observed in this model of disease. In order to clarify whether the attenuated levels of PERK/eIF2α phosphorylation observed in the aged PDK1^K465E/K465E^ mice cortex could have physiological consequences, primary cultures of cortical neurons derived from PDK1^K465E/K465E^ and PDK1^+/+^ mice were treated with tunicamycin, a compound blocking the initial step of glycoprotein biosynthesis that causes the accumulation of unfolded glycoproteins, therefore leading to ER stress. Tunicamycin treatment caused a dramatic up to 90% dose-dependent reduction of cell viability, as assayed with the MTT method, which was followed by a vast up to 5-fold increase in the percentage of cells exhibiting apoptotic nuclear morphology in the control cells. Interestingly, cell viability was two times higher, and the percentage of apoptotic cells 20% reduced, in the PDK1^K465E/K465E^ neurons compared to the controls (Figure 7A). These differences can be attributed to the attenuated tunicamycin-induction of the UPR observed in the PDK1^K465E/K465E^ mutant samples, as denoted by the decreased levels of phospho-PERK Thr890 and phospho-eIF2α Ser-51, but not the BIP, PDI or IRE1α protein levels (Figure 7B).

### Reduced activation of Akt protected the PDK1^K465E/K465E^ neurons against Aβ–induced cell death

**Figure 8:**
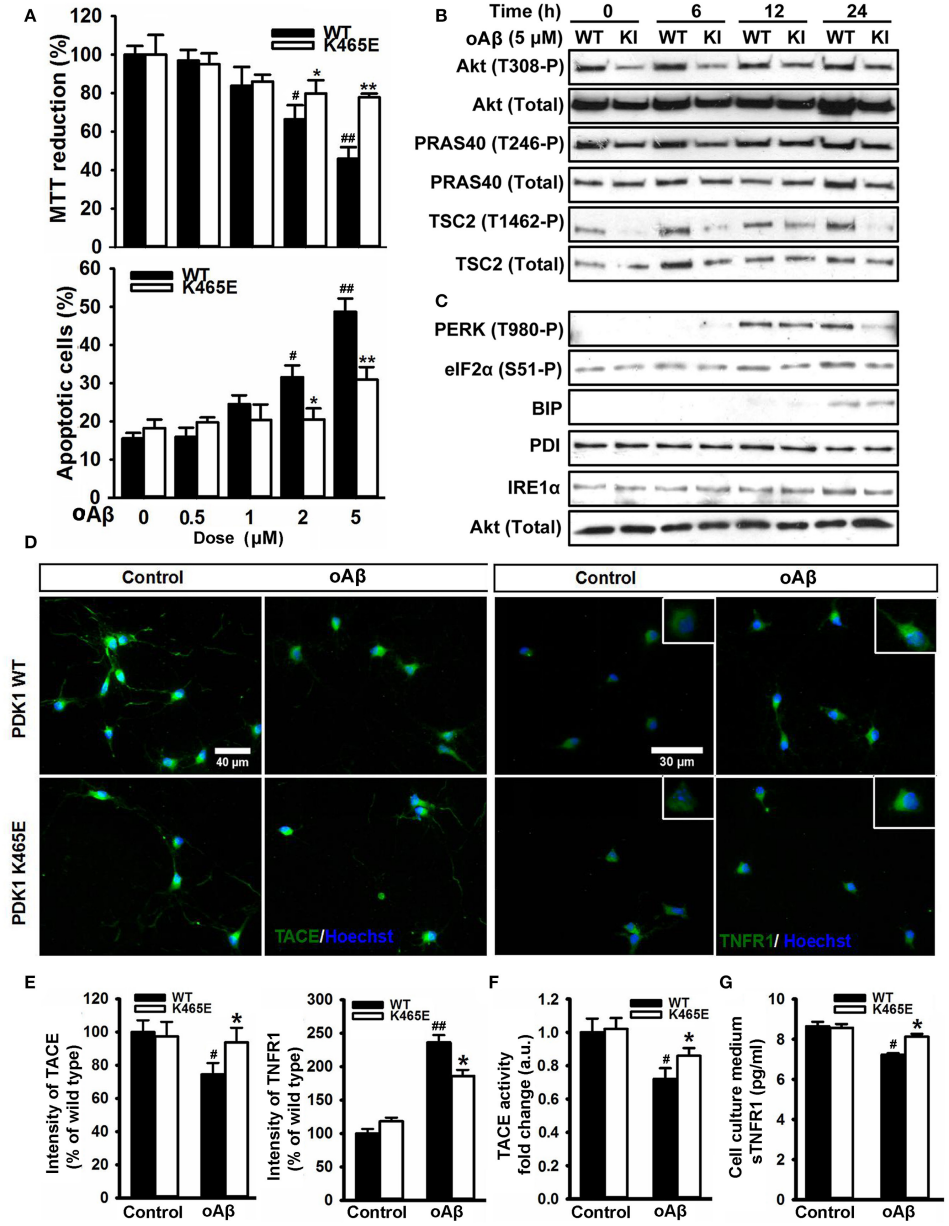
Protection of the PDK1^K465E/K465E^ neurons against Aβ–toxicity. Primary cultures of cortical neurons obtained from PDK1^+/+^ wild type (WT, black bars) and PDK1^K465E/K465E^ mutant (K465E, white bars) E15,5 embryos were grown for 6 days *in vitro* and then treated the indicated concentration of amyloid-β peptides (Aβ) for 48 h **(A)**, with 5 μM of Aβ for the indicated time points in hours **(B,C)**, or with 5 μM of Aβ for 24 h **(D–G)**. **(A)** Upper panel, the cell viability was determined on the indicated cortical cultures with the MTT reduction assay and is expressed as a percentage of the untreated cells; bottom panel, the percentage of apoptotic cells was obtained by scoring the number of nuclei exhibiting chromatin fragmentation divided by the total. Data represents the mean ± SEM for at least five independent embryos per genotype from two separate experiments, with each sample assayed in triplicate. **(B,C)** Protein extracts from matched PDK1 wild-type (WT) and PDK1 mutant knock-in (KI) mice were obtained from the indicated cortical cell samples and then immunoblotted with the depicted antibodies. **(D)** Representative immunofluorescent labeling of TACE or TNFR1 counterstained with the Hoechst nuclear dye, as indicated; bars, 40 and 30 μm for TACE and TNFR1, respectively. **(E)** Quantification of the cell surface signals is expressed as the mean ± SEM for 200 neurons from four different embryos per condition, and expressed as a percentage of the wild type controls. The TACE α-secretase activity **(F)** and the levels of soluble TNFR1 in the culture medium **(G)** were also determined and expressed as the mean ± SEM from four different embryos per condition. ^*^p < 0.05 or ^**^p < 0.005 compared with the corresponding wild types for each condition as obtained by the Student's *t*-test. ^#^*p* < 0.05 and ^##^*p* < 0.005 (Tukey test) statistically significant differences between controls an treatments.

The accumulation of plaques containing misfolded Aβ peptides is a characteristic neuroanatomical hallmark of AD and at the same time a central contributing factor to neurotoxicity. Since the reduced activation of Akt in the PDK1^K465E/K465E^ neurons protected cells against TNFα and ER stress, two proposed key mediators of Aβ-induced pathology, and the PDK1/Akt signaling pathway is hyperactivated in AD transgenic mice models, we reasoned that the PDK1^K465E/K465E^ mutant neurons should be also protected from Aβ-induced neurotoxicity. As expected, the exposure of primary cultures of wild type cortical neurons to Aβ oligomers elicited a dramatic dose-dependent reduction of up to 50% in cell viability, which is accompanied by a massive increase in the percentage of apoptotic cells reaching 50% values at the highest doses of Aβ tested. Remarkably, both the loss of cell viability as well as the increase in the percentage of apoptotic cells are clearly attenuated in the PDK1^K465E/K465E^ neurons, in which a 20% decrease in cell viability and a 30% of apoptotic cells are observed at the highest concentration of Aβ oligomers (Figure 8A). Of note, Aβ treatment did not change the levels of activation of Akt, as judged by the levels of phosphorylation of Akt at the Thr308, PDK1 activation site, as well as the levels of phosphorylation of the Akt substrates PRAS40 at Thr246 and TSC2 at Thr1462, which were as expected consistently reduced in the PDK1^K465E/K465E^ protein extracts compared to the controls along the whole treatment (Figure 8B). By contrast, the exposure of the neurons to Aβ oligomers induced the phosphorylation of PERK at Thr980 and of eIF2α at Ser51 more potently in the PDK1^+/+^ control neurons than in the PDK1^K465E/K465E^ mutant ones; BIP protein was only induced upon 24 h of treatment and to the same level in the two genotypes analyzed, whereas PDI and IRE1α levels remained unchanged (Figure 8C). Of note, Aβ treatment also induced a 20% decrease in the levels of TACE accompanied by a 2.5-fold increase in the levels of membrane-bound TNFR1 in the control cells, which were less pronounced in the PDK1^K465E/K465E^ mutant cells (Figures 8D,E). In agreement with that, the TACE α-secretase catalytic activity (Figure 8F) as well as the levels of soluble TNFR1 released to the medium (Figure 8G) were further 10% reduced in the PDK1^+/+^ wild type cells when compared to the PDK1^K465E/K465E^ mutant cultures.

**Figure 9:**
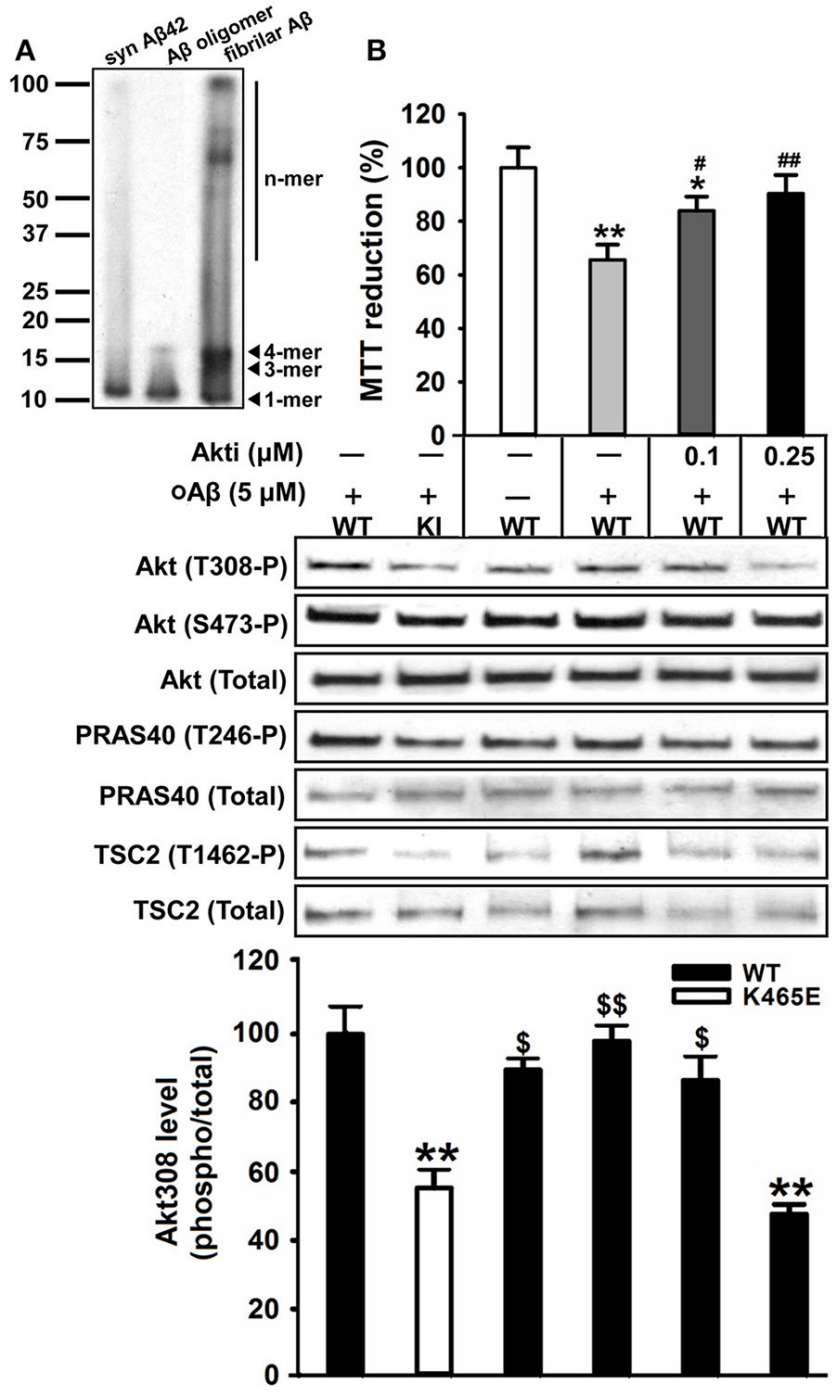
Reducing the levels of Akt activation protected wild type cortical neurons against amyloid-beta induced-toxicity. **(A)** Western blot analysis of the oligomeric stage of the synthetic Aβ peptides preparation (Syn Aβ42), upon addition to the primary neurons (Aβ oligomer), and in the discarded insoluble pellet (fibrillar Aβ). Arrows indicate positions of monomers (1-mer), trimers (3-mer), tetramers (4-mer) and other higher molecular weight forms (n-mer). **(B)** Wild type primary cortical cells were cultured in complete medium for 6 days and then treated in the absence or presence of 5 μM of oligomeric amyloid-β (oAβ) and the indicated concentrations of the Akti-1/2 inhibitor. Upper panel, the cell viability was determined with the MTT reduction assay and is expressed as a percentage of the untreated cells; Middle panel, Protein extracts were obtained from the indicated PDK1 wild-type (WT) and PDK1 mutant knock-in (KI) mice cortical cell samples and then immunoblotted with indicated phospho and total antibodies; band densitometry quantification of the ratio between Akt308 phosphorylation and total protein levels is shown at the bottom, where values are represented as percentage of control. Data represents the mean ± SEM for at least five independent embryos per genotype from two separate experiments, with each sample assayed in triplicate. ^*^p < 0.05 and ^**^p < 0.005 (Student's *t*-test) compared with the untreated controls; ^#^*p* < 0.05 and ^##^*p* < 0.005 (Tukey test) between oAβ plus the inhibitor and oAβ alone; ^$^*p* < 0.05 and ^$$^*p* < 0.005 (Tukey test) between oAβ plus the different concentration inhibitor and the untreated PDK1 mutant samples.

In order to assess the contribution of the Akt signaling to the Aβ-toxicity protection exhibited by the PDK1^K465E/K465E^ mutant cells, we pre-treated wild type cultures with increasing doses of the Akt1 and Akt2 isoform-specific allosteric inhibitor Akti-1/2 prior to the exposure to Aβ oligomers. Pharmacological inhibition of Akt activity to levels equivalent to those found in the PDK1^K465E/K465E^ mutant cells protected wild type cortical cells against Aβ-induced toxicity, thereby mimicking the impact of the PDK1 K465E mutation in impeding Akt activation (Figure 9).

## Discussion

In the present study, we employed the PDK1^K465E/K465E^ knock-mice with reduced Akt activity but otherwise intact PI3K/PDK1 signaling to interrogate the relevance of this pathway to AD. These mice were first generated to explore the importance of the PDK1-PtdIns(3,4,5)P_3_ interaction regarding Akt activation, and were meant to be a unique genetic tool to specifically ablate Akt functions (Bayascas, 2008). Mutation of the PDK1 PH-domain caused a significant reduction in the rate of Akt activation, which was surprisingly still able to be partially phosphorylated and activated by PDK1 through the docking site-dependent mechanism (Najafov et al., 2012; Zhou et al., 2014). We know report that these deficits in Akt signaling tend to be attenuated in an age-dependent manner both in the cortex and the hippocampus, which correlated with elevated concentration of BDNF in both brain areas in the PDK1 mutant mice.

The resultant Akt hypomorphic mice exhibited growth defects and were smaller but otherwise viable, fertile and healthy through the adulthood. Moreover, in spite of the essentiality of Akt in the control of neuronal viability during development (Datta et al., 1999), the neuronal survival responses were still preserved in the PDK1^K465E/K465E^ knock-in mice, which exhibited a normal pattern of cortical layering and connectivity and no signs of brain age-related pathology (Zurashvili et al., 2013).

What is more, PDK1 activity was found increased in the brain samples of individuals with AD, whereas inhibition of PDK1 reduced AD pathology in mice but at the same time resulted in long term toxicity (Pietri et al., 2013). Since the PDK1 L155E mutation abrogating PDK1 signaling with intact Akt activation caused profound alterations in mice (Cordón-Barris et al., 2016), we postulated that inhibition of Akt might have protected mice against AD, whereas inhibition of the docking-site dependent PDK1 substrates might have been responsible for the toxicity of the treatment.

In agreement with that notion, we consistently observed in the cortex and the hippocampus of two different AD transgenic mice models an age-dependent increase in the levels of PDK1 activity, as judged by the elevated levels of auto-phosphorylation at the Ser241 within the PDK1 activation loop as well as the elevated phosphorylation of the downstream Akt substrates. Hyperactive PI3K/Akt signaling has been widely documented in AD mice models (Stein and Johnson, 2002; Sah et al., 2017) and patients (Tramutola et al., 2015). However, in advances stages of the disease, sustained Aβ-mediated activation of Akt caused desensitization of the PI3K pathway to extracellular signals (Moloney et al., 2010; O'Neill et al., 2012; Talbot et al., 2012). Indeed, aberrant activation of components of the insulin signaling pathway, as well as decreased responsiveness to insulin, are commonly found in post-mortem brain samples from patients with AD, which were classified as cerebral insulin resistance (De Felice et al., 2014; Yarchoan and Arnold, 2014; Biessels and Reagan, 2015). Phosphorylation of the Insulin Receptor Substrate-1 (IRS1) by the Akt/mTORC1/S6K negative feedback axis plays a primary role in uncoupling PI3K to a number of tyrosine kinase receptors (Shah et al., 2004; O'Neill, 2013). Increased phosphorylation of Akt at Ser473 was also observed in the aged AD transgenic mice mutant samples which paralleled that of Akt Thr308, the PDK1 site. In contrast, the deficient activation of Akt observed in the PDK1^K465E/K465E^ mice arise from inefficient PDK1-mediated phosphorylation of Akt at Thr308 with intact Akt phosphorylation at Ser473, mTORC2 site. Therefore, a role for mTORC2 hyperactivity in mediating Aβ-mediated pathology can be envisaged. In this regard, Aβ was previously shown to induce mTORC1 hyperactivity through Akt-mediated phosphorylation of PRAS40 in 3xTg-AD mice (Caccamo et al., 2011). Indeed, we show increased PRAS40 Thr246 phosphorylation both in APP/PS1 and 3xTg-AD old mice.

The PI3K/Akt signaling pathway is also antagonized by JNK-mediated phosphorylation of IRS1 in response to inflammatory factors such as TNFα (Tanti and Jager, 2009). In AD, microglia activation and pro-inflammatory cytokines production leads to tau hyperphosphorylation and Aβ accumulation, which in turn increases the release of these cytokines (Wang et al., 2015). We found the production of three pro-inflammatory cytokines, IL-2, IL-9 and TNFα, attenuated in the aged PDK1^K465E/K465E^ mutant mice. Higher serum TNFα concentrations have been reported in AD and dementia (Alvarez et al., 1996; Bruunsgaard et al., 1999). Opposite to the PDK1^K465E/K465E^ aged mice, IL-9 levels are significantly augmented in AD patients (Dardalhon et al., 2008), whereas the implication of IL-2 in AD has not been documented.

The TACE secretase, which is primarily known for its role in promoting the shedding of cell surface–bound TNFα and its receptors (Gooz, 2010), mediates also the α-cleavage of the amyloid precursor protein thereby precluding Aβ production (Buxbaum et al., 1998). In AD patients, the concentration of the soluble forms of APPα and TNFR1 in the cerebrospinal fluid are decreased due to reduced brain TACE α-secretase activity (Sennvik et al., 2000; Yao et al., 2015). Moreover, inhibition of PDK1 restored TACE α-secretase activity and cleavage of APP and TNFR1 in neurons affected by Aβ deposition by preventing the phosphorylation and internalization of TACE (Pietri et al., 2013). In accordance with these observations, we found that while the TACE α-secretase activity and the levels of sTNFR1 were decreased with aging in brain extracts from the PDK1^+/+^ control mice, they were significantly higher at 16 and 21 months of age in the PDK1^K465E/K465E^ mice brain when compared to age-matched controls. This was accompanied by increased TACE with decreased unshedded TNFR1 accumulation at the plasma membrane of the aged PDK1^K465E/K465E^ mice brain, which correlated with reduced phosphorylation of TACE at Thr sites in the mutant samples. Using TNFR1 shedding as a readout of TACE activity, we found that the membrane-associated TACE α-secretase activity and the concentration of sTNFR1 were significantly higher, whilst the levels of TNFR1 at the cell surface lower, in the PDK1^K465E/K465E^ neuronal cells treated with Aβ or TNFα compared with the PDK1^+/+^ wild type cells, thereby pointing out to a prominent role of Akt downstream of PDK1 in mediating TACE downregulation induced by Aβ and other inflammatory factors.

Decreased TNFR1 levels at the cell surface should render the PDK1 mutant cells less vulnerable to soluble TNFα-toxicity. Nevertheless, TNFα only compromised cell viability in the PDK1^+/+^ wild type cultures in the presence of the RNA synthesis inhibitor Actinomycin-D, and to a lesser extent in the PDK1^K465E/K465E^ mutant cultures. Some studies indicate that TNFα is a prosurvival factor for neurons (Cheng et al., 1994; Courtney et al., 1997), that NF-kappaB is not activated by TNFα in neurons (Jarosinski et al., 2001; Mao et al., 2006; Listwak et al., 2013), and that the TNFR2 may as well be responsible for TNFα-induced toxicity (Gary et al., 1998). TNFR1 signaling cascade is required for Aβ-induced neuronal cell death (Li et al., 2004); conversely, TNFR1 is required for Aβ generation (He et al., 2007). In contrast to TNFα, the neurotoxicity elicited by Aβ peptides was found dramatically attenuated in the PDK1^K465E/K465E^ neurons compared to the PDK1^+/+^ controls. That prompted us to browse for more downstream cellular mechanisms that could explain the differential sensitivity of the PDK1^K465E/K465E^ mutant cells to Aβ-related toxicity.

Among them, the UPR is a protective cellular response induced during periods ER stress, which reduces the unfolded protein load and returns the protein-folding homeostasis (Halliday and Mallucci, 2015). In AD, the aberrant proteostasis of Aβ and Tau induces the UPR and contributes to exacerbate the neuronal cell loss (Hetz and Mollereau, 2014). For example, phosphorylation of eIF2α is elevated in the brain of AD patients and AD mice models, and inhibition of the upstream kinase PERK prevent deficits in protein synthesis, synaptic plasticity and spatial memory in APP/PS1 mice (Ma et al., 2013). In agreement with that notion, we found the levels of phosphorylation of both PERK at Thr980 and eIF2α at Ser51 markedly increased in the cortex of the 12-months old 3xTg-AD mice, whereas the protein levels of other components of the UPR analyzed such as BIP, PDI, IRE1α or CHOP remained unchanged. In sharp contrast, phosphorylation of PERK at Thr980 and eIF2α at Ser51 were reduced in the old PDK1^K465E/K465E^ mutant mice or in mutant cortical cultures treated with tunicamycin or Aβ compared with the PDK1^+/+^ wild type mice and cells. Attenuated Akt signaling allows the FOXO-mediated transcription of genes counteracting age-related proteotoxicity in AD mice (Cohen et al., 2009; Douglas and Dillin, 2010). Moreover, we found diminished number of apoptotic cells induced by tunicamycin, which were more robustly attenuated upon Aβ treatment in the PDK1^K465E/K465E^ neurons compared with the PDK1^+/+^ wild type controls, especially when they were used at highest doses. Therefore, the inefficient binding of PDK1 to PtdIns(3,4,5)P_3_ in the PDK1^K465E/K465E^ mice leading to reduced phosphorylation of Akt at Thr308 and hypoactivation of the downstream signaling pathway seems to play an important role in attenuating the UPR in an age-dependent manner. These observations point to an Akt-dependent activation of the PERK/eIF2α pathway with aging, which was however previously shown to phosphorylate and inhibit PERK in cells exposed to ER stress or oxidative stress (Mounir et al., 2011). All the above findings raise the possibility of the existence of a positive feedback effect of Akt on the PERK-eIF2α axis activation under an unbalance of the ER stress, which might be attenuated in the PDK1^K465E/K465E^ mice, leading to a reduction in the neuronal cell death response. In this regard, the ER stress is proposed to contribute to the development of brain insulin resistance (Fu et al., 2012). PKR, the dsRNA-dependent protein kinase, functional homolog of PERK, has been also shown to mediate brain IRS1 inhibition and elF2α phosphorylation induced by Aβ and TNFα in mice and monkeys (Lourenco et al., 2013). Moreover, suppression of elF2α phosphorylation provides neuroprotection in mouse models of AD (Ma et al., 2013). This mechanism, together with the upregulation of TACE and shedding of APP and TNFR1, could synergistically account for the Aβ-resistance of the PDK1^K465E/K465E^ neurons, which could be mimicked upon treatment with the Akti-1/2 compound inhibiting Akt1 and Akt2, but not Akt3, the isoform whose levels are highly and specifically enriched in brain tissues. Of note, the compound employed have nowadays been developed into an orally available drug approved for human clinical trial in patients with advanced solid tumors (Yap et al., 2011) and there are currently 217 clinical trials listed on the NIH clinical trials website (ClinicalTrials.gov) to evaluate the therapeutic efficacy of Akt inhibitors for the treatment of cancer. Our results provide genetic construct validity for Akt inhibition as a promising therapy in the treatment of AD.

## Conclusion

We observed age-dependent hyperactivation of the PDK1/Akt signaling pathway in both the double APP/PS1 and triple 3xTg-AD transgenic mice models of AD, and employed the PDK1^K465E/K465E^ mice with reduced Akt activity to interrogate the relevance of this observation. We demonstrated how the levels of Akt signaling dictate diverse outcomes regarding TACE activation, TNFR1 processing and UPR actions, which determine the sensitivity of the neurons to TNFα, ER stress and ultimately Aβ toxicity. These findings suggest that the PDK1^K465E/K465E^ mice might be protected against AD, and provide genetic validation of Akt as a new therapeutical target for the treatment of this disease.

## Author contributions

The conception and design of the work was done by JB, OA, VC, and VY. Experimental acquisition of data was done by SY, RP, SP-G, MB, and JP, whilst analysis and interpretation of data were done by all the authors. Drafting of the manuscript was done by JB. All the authors revised the manuscript critically for important intellectual content. All the authors contributed significantly to the latter version of the manuscript and approved the final version of the manuscript.

### Conflict of interest statement

The authors declare that the research was conducted in the absence of any commercial or financial relationships that could be construed as a potential conflict of interest. The reviewer AD and handling Editor declared their shared affiliation.

## References

[B1] Alessi D. R. James S. R. Downes C. P. Holmes A. B. Gaffney P. R. Reese C. B.. (1997). Characterization of a 3-phosphoinositide-dependent protein kinase which phosphorylates and activates protein kinase Balpha. Curr. Biol. 7, 261–269. 10.1016/S0960-9822(06)00122-9 9094314

[B2] Alvarez X. A. Franco A. Fernández-Novoa L. Cacabelos R. (1996). Blood levels of histamine, IL-1 beta, and TNF-alpha in patients with mild to moderate Alzheimer disease. Mol. Chem. Neuropathol. 29, 237–252. 10.1007/BF02815005 8971699

[B3] Angelucci F. Spalletta G. di Iulio F. Ciaramella A. Salani F. Colantoni L. (2009). Alzheimer'S disease (ad) and mild cognitive impairment (mci) patients are characterized by increased bdnf serum levels. Alzheimer's Dement. 5, P272–P273. 10.1016/j.jalz.2009.04.346 20205668

[B4] Bartke A. (2008). Impact of reduced insulin-like growth factor-1/insulin signaling on aging in mammals: novel findings. Aging Cell 7, 285–290. 10.1111/j.1474-9726.2008.00387.x 18346217

[B5] Bayascas J. R. (2008). Dissecting the role of the 3-phosphoinositide-dependent protein kinase-1 (PDK1) signalling pathways. Cell Cycle 7, 2978–2982. 10.4161/cc.7.19.6810 18802401

[B6] Bayascas J. R. (2010). PDK1: The major transducer of PI 3-kinase actions. Curr. Top. Microbiol. Immunol. 346, 9–29. 10.1007/82_2010_43 20563709

[B7] Bayascas J. R. Sakamoto K. Armit L. Arthur J. S. C. Alessi D. R. (2006). Evaluation of approaches to generation of tissue-specific knock-in mice. J. Biol. Chem. 281, 28772–28781. 10.1074/jbc.M606789200 16887794

[B8] Bayascas J. R. Wullschleger S. Sakamoto K. Garcia-Martinez J. M. Clacher C. Komander D.. (2008). Mutation of the PDK1 PH Domain Inhibits Protein Kinase B/Akt, Leading to Small Size and Insulin Resistance. Mol. Cell. Biol. 28, 3258–3272. 10.1128/MCB.02032-07 18347057 PMC2423167

[B9] Biessels G. J. Reagan L. P. (2015). Hippocampal insulin resistance and cognitive dysfunction. Nat. Rev. Neurosci. 16, 660–671. 10.1038/nrn4019 26462756

[B10] Biondi R. M. Komander D. Thomas C. C. Lizcano J. M. Deak M. Alessi D. R.. (2002). High resolution crystal structure of the human PDK1 catalytic domain defines the regulatory phosphopeptide docking site. EMBO J. 21, 4219–4228. 10.1093/emboj/cdf437 12169624 PMC126174

[B11] Bruunsgaard H. Andersen-Ranberg K. Jeune B. Pedersen A. N. Skinhøj P. Pedersen B. K. (1999). A high plasma concentration of TNF-alpha is associated with dementia in centenarians. J. Gerontol. A. Biol. Sci. Med. Sci. 54, M357–M364. 10.1093/gerona/54.7.M357 10462168

[B12] Buxbaum J. D. Liu K. N. Luo Y. Slack J. L. Stocking K. L. Peschon J. J.. (1998). Evidence that tumor necrosis factor alpha converting enzyme is involved in regulated alpha-secretase cleavage of the Alzheimer amyloid protein precursor. J. Biol. Chem. 273, 27765–27767. 10.1074/jbc.273.43.27765 9774383

[B13] Caccamo A. Maldonado M. A. Majumder S. Medina D. X. Holbein W. Magrí A.. (2011). Naturally secreted amyloid-β increases mammalian target of rapamycin (mTOR) activity via a PRAS40-mediated mechanism. J. Biol. Chem. 286, 8924–8932. 10.1074/jbc.M110.180638 21266573 PMC3058958

[B14] Cheng B. Christakos S. Mattson M. P. (1994). Tumor necrosis factors protect neurons against metabolic-excitotoxic insults and promote maintenance of calcium homeostasis. Neuron 12, 139–153. 10.1016/0896-6273(94)90159-7 7507336

[B15] Cohen E. Paulsson J. F. Blinder P. Burstyn-Cohen T. Du D. Estepa G.. (2009). Reduced IGF-1 signaling delays age-associated proteotoxicity in mice. Cell 139, 1157–1169. 10.1016/j.cell.2009.11.014 20005808 PMC3017511

[B16] Collins B. J. Deak M. Arthur J. S. Armit L. J. Alessi D. R. (2003). *In vivo* role of the PIF-binding docking site of PDK1 defined by knock-in mutation. EMBO J. 22, 4202–4211. 10.1093/emboj/cdg407 12912918 PMC175797

[B17] Cordón-Barris L. Pascual-Guiral S. Yang S. Giménez-Llort L. Lope-Piedrafita S. Niemeyer C. (2016). Mutation of the 3-phosphoinositide-dependent protein kinase-1 (PDK1) substrate-docking site in the developing brain causes microcephaly with abnormal brain morphogenesis independently of Akt, leading to impaired cognition and disruptive behaviors. Mol. Cell. Biol. 36, 2967–2982. 10.1128/MCB.00230-16 PMC5108884 27644329

[B18] Courtney M. J. Akerman K. E. Coffey E. T. (1997). Neurotrophins protect cultured cerebellar granule neurons against the early phase of cell death by a two-component mechanism. J. Neurosci. 17, 4201–4211. 9151737 10.1523/JNEUROSCI.17-11-04201.1997 PMC6573533

[B19] Cowan C. M. Bossing T. Page A. Shepherd D. Mudher A. (2010). Soluble hyper-phosphorylated tau causes microtubule breakdown and functionally compromises normal tau *in vivo*. Acta Neuropathol. 120, 593–604. 10.1007/s00401-010-0716-8 20617325

[B20] Dardalhon V. Awasthi A. Kwon H. Galileos G. Gao W. Sobel R. A.. (2008). IL-4 inhibits TGF-beta-induced Foxp3+ T cells and, together with TGF-beta, generates IL-9+ IL-10+ Foxp3(-) effector T cells. Nat. Immunol. 9, 1347–1355. 10.1038/ni.1677 18997793 PMC2999006

[B21] Datta S. R. Brunet A. Greenberg M. E. (1999). Cellular survival: a play in three Akts. Genes Dev. 13, 2905–2927. 10.1101/gad.13.22.2905 10579998

[B22] De Felice F. G. Ferreira S. T. Ott A. Stolk R. Harskamp F. van Pols H.. (2014). Inflammation, defective insulin signaling, and mitochondrial dysfunction as common molecular denominators connecting type 2 diabetes to Alzheimer disease. Diabetes 63, 2262–2272. 10.2337/db13-1954 24931033

[B23] Douglas P. M. Dillin A. (2010). Protein homeostasis and aging in neurodegeneration. J. Cell Biol. 190, 719–729. 10.1083/jcb.201005144 20819932 PMC2935559

[B24] Edwards D. R. Handsley M. M. Pennington C. J. (2009). The ADAM metalloproteinases. Mol. Aspects Med. 29, 258–289. 10.1016/j.mam.2008.08.001 18762209 PMC7112278

[B25] Engelman J. A. Luo J. Cantley L. C. (2006). The evolution of phosphatidylinositol 3-kinases as regulators of growth and metabolism. Nat. Rev. Genet. 7, 606–619. 10.1038/nrg1879 16847462

[B26] Fontana L. Partridge L. Longo V. D. (2010). Extending healthy life span–from yeast to humans. Science 328, 321–326. 10.1126/science.1172539 20395504 PMC3607354

[B27] Foukas L. C. Withers D. J. (2010). Phosphoinositide signalling pathways in metabolic regulation. Curr. Top. Microbiol. Immunol. 346, 115–141. 10.1007/82_2010_59 20517721

[B28] Fu S. Watkins S. M. Hotamisligil G. S. (2012). The role of endoplasmic reticulum in hepatic lipid homeostasis and stress signaling. Cell Metab. 15, 623–634. 10.1016/j.cmet.2012.03.007 22560215

[B29] Gary D. S. Bruce-Keller A. J. Kindy M. S. Mattson M. P. (1998). Ischemic and excitotoxic brain injury is enhanced in mice lacking the p55 tumor necrosis factor receptor. J. Cereb. Blood Flow Metab. 18, 1283–1287. 10.1097/00004647-199812000-00001 9850139

[B30] Gooz M. (2010). ADAM-17: the enzyme that does it all. Crit. Rev. Biochem. Mol. Biol. 45, 146–169. 10.3109/10409231003628015 20184396 PMC2841225

[B31] Gozzelino R. Sole C. Llecha N. Segura M. F. Moubarak R. S. Iglesias-Guimarais V.. (2008). BCL-XL regulates TNF-alpha-mediated cell death independently of NF-kappaB, FLIP and IAPs. Cell Res. 18, 1020–1036. 10.1038/cr.2008.76 18591962

[B32] Griffin R. J. Moloney A. Kelliher M. Johnston J. A. Ravid R. Dockery P.. (2005). Activation of Akt/PKB, increased phosphorylation of Akt substrates and loss and altered distribution of Akt and PTEN are features of Alzheimer's disease pathology. J. Neurochem. 93, 105–117. 10.1111/j.1471-4159.2004.02949.x 15773910

[B33] Halliday M. Mallucci G. R. (2015). Review: modulating the unfolded protein response to prevent neurodegeneration and enhance memory. Neuropathol. Appl. Neurobiol. 41, 414–427. 10.1111/nan.12211 25556298 PMC5053297

[B34] He P. Zhong Z. Lindholm K. Berning L. Lee W. Lemere C.. (2007). Deletion of tumor necrosis factor death receptor inhibits amyloid β generation and prevents learning and memory deficits in Alzheimer's mice. J. Cell Biol. 178, 829–841. 10.1083/jcb.200705042 17724122 PMC2064547

[B35] Hetz C. Mollereau B. (2014). Disturbance of endoplasmic reticulum proteostasis in neurodegenerative diseases. Nat. Rev. Neurosci. 15, 233–249. 10.1038/nrn3689 24619348

[B36] Holcomb L. Gordon M. N. McGowan E. Yu X. Benkovic S. Jantzen P.. (1998). Accelerated Alzheimer-type phenotype in transgenic mice carrying both mutant amyloid precursor protein and presenilin 1 transgenes. Nat. Med. 4, 97–100. 10.1038/nm0198-097 9427614

[B37] Huang Y. Mucke L. (2012). Alzheimer mechanisms and therapeutic strategies. Cell 148, 1204–1222. 10.1016/j.cell.2012.02.040 22424230 PMC3319071

[B38] Jarosinski K. W. Whitney L. W. Massa P. T. (2001). Specific deficiency in nuclear factor-kappaB activation in neurons of the central nervous system. Lab. Invest. 81, 1275–1288. 10.1038/labinvest.3780341 11555675

[B39] Kilkenny C. Browne W. J. Cuthill I. C. Emerson M. Altman D. G. (2010). Improving bioscience research reporting: The ARRIVE guidelines for reporting animal research. J. Pharmacol. Pharmacother. 1, 94–99. 10.4103/0976-500X.72351 21350617 PMC3043335

[B40] Leslie N. R. Downes C. P. (2004). PTEN function: how normal cells control it and tumour cells lose it. Biochem. J. 382, 1–11. 10.1042/BJ20040825 15193142 PMC1133909

[B41] Li R. Yang L. Lindholm K. Konishi Y. Yue X. Hampel H.. (2004). Tumor necrosis factor death receptor signaling cascade is required for amyloid-beta protein-induced neuron death. J. Neurosci. 24, 1760–1771. 10.1523/JNEUROSCI.4580-03.2004 14973251 PMC6730458

[B42] Lippens G. Sillen A. Landrieu I. Amniai L. Sibille N. Barbier P. (2007). Tau aggregation in Alzheimer' s disease what role for phosphorylation? Prion 1, 21–25. 10.4161/pri.1.1.4055 19164903 PMC2633703

[B43] Listwak S. J. Rathore P. Herkenham M. (2013). Minimal NF-κB activity in neurons. Neuroscience 250, 282–299. 10.1016/j.neuroscience.2013.07.013 23872390 PMC3785079

[B44] Lourenco M. V. Clarke J. R. Frozza R. L. Bomfim T. R. Forny-Germano L. Batista A. F.. (2013). TNF-α mediates PKR-dependent memory impairment and brain IRS-1 inhibition induced by Alzheimer's β-amyloid oligomers in mice and monkeys. Cell Metab. 18, 831–843. 10.1016/j.cmet.2013.11.002 24315369

[B45] Ma T. Trinh M. A. Wexler A. J. Bourbon C. Gatti E. Pierre P. (2013). Suppression of eIF2a kinases alleviates Alzheimer's disease-related plasticity and memory deficits. Nat. Neurosci. 16, 1299–1305. 10.1038/nn.3486 23933749 PMC3756900

[B46] Manterola L. Hernando-Rodriguez M. Ruiz A. Apraiz A. Arrizabalaga O. Vellon L.. (2013). 1-42 beta-amyloid peptide requires PDK1/nPKC/Rac 1 pathway to induce neuronal death. Transl. Psychiatry 3:e219. 10.1038/tp.2012.147 23340502 PMC3566727

[B47] Mao X. Moerman-Herzog A. M. Wang W. Barger S. W. (2006). Differential transcriptional control of the superoxide dismutase-2 κB element in neurons and astrocytes. J. Biol. Chem. 281, 35863–35872. 10.1074/jbc.M604166200 17023425 PMC2063448

[B48] Mercado G. Valdes P. Hetz C. (2013). An ERcentric view of Parkinson's disease. Trends Mol. Med. 19, 166–175. 10.1016/j.molmed.2012.12.005 23352769

[B49] Moloney A. M. Griffin R. J. Timmons S. O'Connor R. Ravid R. O'Neill C. (2010). Defects in IGF-1 receptor, insulin receptor and IRS-1/2 in Alzheimer's disease indicate possible resistance to IGF-1 and insulin signalling. Neurobiol. Aging 31, 224–243. 10.1016/j.neurobiolaging.2008.04.002 18479783

[B50] Mora A. Komander D. Van Aalten D. M. Alessi D. R. (2004). PDK1, the master regulator of AGC kinase signal transduction. Semin. Dev.Biol. 15, 161–170. 10.1016/j.semcdb.2003.12.022 15209375

[B51] Mounir Z. Krishnamoorthy J. L. Wang S. Papadopoulou B. Campbell S. Muller W. J. (2011). Akt determines cell fate through inhibition of the PERK-eIF2a phosphorylation pathway. Sci. Signal. 4:ra62 10.1126/scisignal.2001630 21954288 PMC3752779

[B52] Najafov A. Shpiro N. Alessi D. R. (2012). Akt is efficiently activated by PIF-pocket- and PtdIns(3,4,5)P3-dependent mechanisms leading to resistance to PDK1 inhibitors. Biochem. J. 448, 285–295. 10.1042/BJ20121287 23030823

[B53] O'Neill C. (2013). PI3-kinase/Akt/mTOR signaling: impaired on/off switches in aging, cognitive decline and Alzheimer's disease. Exp. Gerontol. 48, 647–653. 10.1016/j.exger.2013.02.025 23470275

[B54] O'Neill C. Kiely A. P. Coakley M. F. Manning S. Long-Smith C. M. (2012). Insulin and IGF-1 signalling: longevity, protein homoeostasis and Alzheimer's disease. Biochem. Soc. Trans. 40, 721–727. 10.1042/BST20120080 22817723

[B55] Oddo S. Caccamo A. Shepherd J. D. Murphy M. P. Golde T. E. Kayed R.. (2003). Triple-transgenic model of Alzheimer's disease with plaques and tangles: intracellular Aβ and synaptic dysfunction. Neuron 39, 409–421. 10.1016/S0896-6273(03)00434-3 12895417

[B56] Oddo S. Caccamo A. Tseng B. Cheng D. Vasilevko V. Cribbs D. H.. (2008). Blocking Abeta42 accumulation delays the onset and progression of tau pathology via the C terminus of heat shock protein70-interacting protein: a mechanistic link between Abeta and tau pathology. J. Neurosci. 28, 12163–12175. 10.1523/JNEUROSCI.2464-08.2008 19020010 PMC6671718

[B57] Ortega-Molina A. Efeyan A. Lopez-Guadamillas E. Muñoz-Martin M. Gomez-Lopez G. Cañamero M.. (2012). Pten positively regulates brown adipose function, energy expenditure, and longevity. Cell Metab. 15, 382–394. 10.1016/j.cmet.2012.02.001 22405073

[B58] Pei J.-J. Khatoon S. An W.-L. Nordlinder M. Tanaka T. Braak H.. (2003). Role of protein kinase B in Alzheimer's neurofibrillary pathology. Acta Neuropathol. 105, 381–392. 10.1007/s00401-002-0657-y 12624792

[B59] Phillips H. S. Hains J. M. Armanini M. Laramee G. R. Johnson S. A. Winslow J. W. (1991). BDNF mRNA is decreased in the hippocampus of individuals with Alzheimer's disease. Neuron 7, 695–702. 10.1016/0896-6273(91)90273-3 1742020

[B60] Pietri M. Dakowski C. Hannaoui S. leaume-Butaux A. Hernandez-Rapp J. Ragagnin A.. (2013). PDK1 decreases TACE-mediated alpha-secretase activity and promotes disease progression in prion and Alzheimer's diseases. Nat. Med. 19, 1124–1131. 10.1038/nm.3302 23955714

[B61] Rickle A. Bogdanovic N. Volkman I. Winblad B. Ravid R. Cowburn R. F. (2004). Akt activity in Alzheimer's disease and other neurodegenerative disorders. Neuroreport 15, 955–959. 10.1097/00001756-200404290-00005 15076714

[B62] Ryder J. Su Y. Ni B. (2004). Akt/GSK3β serine/threonine kinases: evidence for a signalling pathway mediated by familial Alzheimer's disease mutations. Cell. Signal. 16, 187–200. 10.1016/j.cellsig.2003.07.004 14636889

[B63] Sah S. K. Lee C. Jang J.-H. Park G. H. (2017). Effect of high-fat diet on cognitive impairment in triple-transgenic mice model of Alzheimer's disease. Biochem. Biophys. Res. Commun. 493, 731–736. 10.1016/j.bbrc.2017.08.122 28865961

[B64] Sennvik K. Fastbom J. Blomberg M. Wahlund L. O. Winblad B. Benedikz E. (2000). Levels of α- and β-secretase cleaved amyloid precursor protein in the cerebrospinal fluid of Alzheimer's disease patients. Neurosci. Lett. 278, 169–172. 10.1016/S0304-3940(99)00929-5 10653020

[B65] Shah O. Wang Z. Hunter T. (2004). Inappropriate activation of the TSC/Rheb/mTOR/S6K Cassette Induces IRS1/2 depletion, insulin resistance, and cell survival deficiencies. Curr. Biol. 14, 1650–1656. 10.1016/j.cub.2004.08.026 15380067

[B66] Stein T. D. Johnson J. A. (2002). Lack of neurodegeneration in transgenic mice overexpressing mutant amyloid precursor protein is associated with increased levels of transthyretin and the activation of cell survival pathways. J. Neurosci. 22, 7380–7388. 12196559 10.1523/JNEUROSCI.22-17-07380.2002 PMC6758007

[B67] Talbot K. Wang H. Y. Kazi H. Han L. Y. Bakshi K. P. Stucky A.. (2012). Demonstrated brain insulin resistance in Alzheimer's disease patients is associated with IGF-1 resistance, IRS-1 dysregulation, and cognitive decline. J. Clin. Invest. 122, 1316–1338. 10.1172/JCI59903 22476197 PMC3314463

[B68] Tanti J. F. Jager J. (2009). Cellular mechanisms of insulin resistance: role of stress-regulated serine kinases and insulin receptor substrates (IRS) serine phosphorylation. Curr. Opin. Pharmacol. 9, 753–762. 10.1016/j.coph.2009.07.004 19683471

[B69] Tramutola A. Triplett J. C. Di Domenico F. Niedowicz D. M. Murphy M. P. Coccia R.. (2015). Alteration of mTOR signaling occurs early in the progression of Alzheimer disease (AD): analysis of brain from subjects with pre-clinical AD, amnestic mild cognitive impairment and late-stage AD. J. Neurochem. 133, 739–749. 10.1111/jnc.13037 25645581

[B70] Vanhaesebroeck B. Leevers S. J. Ahmadi K. Timms J. Katso R. Driscoll P. C.. (2001). Synthesis and function of 3-phosphorylated inositol lipids. Annu. Rev. Biochem. 70, 535–602. 10.1146/annurev.biochem.70.1.535 11395417

[B71] Vanhaesebroeck B. Whitehead M. A. Piñeiro R. (2016). Molecules in medicine mini-review: isoforms of PI3K in biology and disease. J. Mol. Med. 94, 5–11. 10.1007/s00109-015-1352-5 26658520

[B72] Wang W.-Y. Tan M.-S. Yu J.-T. Tan L. (2015). Role of pro-inflammatory cytokines released from microglia in Alzheimer's disease. Ann. Transl. Med. 3:136. 10.3978/j.issn.2305-5839.2015.03.49 26207229 PMC4486922

[B73] Wong K.-K. Engelman J. A. Cantley L. C. (2010). Targeting the PI3K signaling pathway in cancer. Curr. Opin. Genet. Dev. 20, 87–90. 10.1016/j.gde.2009.11.002 20006486 PMC2822054

[B74] Wullschleger S. Sakamoto K. Johnstone L. Duce S. Fleming S. Alessi D. R. (2011). How moderate changes in Akt T-loop phosphorylation impact on tumorigenesis and insulin resistance. Dis. Model. Mech. 4, 95–103. 10.1242/dmm.005603 20959631 PMC3008965

[B75] Yang L. Lindholm K. Konishi Y. Li R. Shen Y. (2002). Target depletion of distinct tumor necrosis factor receptor subtypes reveals hippocampal neuron death and survival through different signal transduction pathways. J. Neurosci. 22, 3025–3032. 11943805 10.1523/JNEUROSCI.22-08-03025.2002 PMC6757531

[B76] Yao X.-Q. Jiao S.-S. Saadipour K. Zeng F. Wang Q.-H. Zhu C.. (2015). p75NTR ectodomain is a physiological neuroprotective molecule against amyloid-beta toxicity in the brain of Alzheimer's disease. Mol. Psychiatry 20, 1301–1310. 10.1038/mp.2015.49 25917367 PMC4759103

[B77] Yap T. A. Yan L. Patnaik A. Fearen I. Olmos D. Papadopoulos K.. (2011). First-in-man clinical trial of the oral pan-AKT inhibitor MK-2206 in patients with advanced solid tumors. J. Clin. Oncol. 29, 4688–4695. 10.1200/JCO.2011.35.5263 22025163

[B78] Yarchoan M. Arnold S. E. (2014). Repurposing diabetes drugs for brain insulin resistance in Alzheimer disease. Diabetes 53, 2253–2261. 10.2337/db14-0287 PMC4066335 24931035

[B79] Zhou X. Cordon-Barris L. Zurashvili T. Bayascas J. R. (2014). Fine-tuning the intensity of the PKB/Akt signal enables diverse physiological responses. Cell Cycle 13, 3164–3168. 10.4161/15384101.2014.962954 25485494 PMC4614834

[B80] Zurashvili T. Cordón-Barris L. Ruiz-Babot G. Zhou X. Lizcano J. M. Gómez N.. (2013). Interaction of PDK1 with phosphoinositides is essential for neuronal differentiation but dispensable for neuronal survival. Mol. Cell. Biol. 33:1027. 10.1128/MCB.01052-12 23275438 PMC3623085

