# Supplementary material for: Reducing the Levels of Akt Activation by PDK1 Knock-in Mutation Protects Neuronal Cultures against Synthetic Amyloid-Beta Peptides
Source: Front Aging Neurosci. 2018 Jan 8;9:435. doi: 10.3389/fnagi.2017.00435 (PMC5766684; doi:10.3389/fnagi.2017.00435)
Supplement: Supplementary file 1 [file Table1.docx]

**Reducing the Levels of Akt Activation by PDK1 Knock-in Mutation in Mice Protects neuronal Cultures Against Synthetic Amyloid-Beta peptides treatment.** Shaobin Yang^1^, Sònia Pascual-Guiral^1^, Rebeca Ponce^1^, Lydia Giménez-Llort^2^, María A. Baltrons^3^, Ottavio Arancio^4^, Jose R. Palacio^5^, Victoria Clos^6^, Victor J. Yuste^1^ & Jose R. Bayascas^1*^

**Supplementary Figure 1.** **Hyperactivation of the PDK1/Akt signaling axis in the APP/PS1** **mice brain.**

Cortex protein extracts obtained from wild type (WT) and double transgenic AD mice model (APP/PS1) at 6 months (A) and 12 months (B) of age were subjected to immunoblot analysis with the indicated phospho and total antibodies. Each lane corresponds to a sample derived from a different mouse. Band densitometry quantification of the ratio between phosphorylated and total protein levels represented as percentage of the 6-months-old wild type controls is shown below the panels. The data are represented as the mean ± SEM for at least three different mice per genotype. * *p* < 0.05 and ** *p* < 0.005 compared to controls as obtained by the Student' s *t* test. #*p* < 0.05 (Tukey test) statistically significant difference compared to six months transgenic mice.

**Supplementary Figure 2. Lifetime serum profiling of cytokine and chemokine levels in the PDK1^K465E/K465E^ mutant mice.** The concentration of 24 different cytokines and chemokines was measured by using multiplex methodology on serum samples from PDK1^+/+^ wild type (WT, black bars) and PDK1^K465E/K465E^ mutant (K465E, white bars) mice at the indicated age in months. The data are represented as the mean ± SEM for at least three different mice per genotype. * *p* < 0.05 and ** *p* < 0.005 compared with wild types as obtained by the Student' s *t* test.
