# Supplementary figures and images for: Reducing the Levels of Akt Activation by PDK1 Knock-in Mutation Protects Neuronal Cultures against Synthetic Amyloid-Beta Peptides
Source: Front Aging Neurosci. 2018 Jan 8;9:435. doi: 10.3389/fnagi.2017.00435 (PMC5766684; doi:10.3389/fnagi.2017.00435)

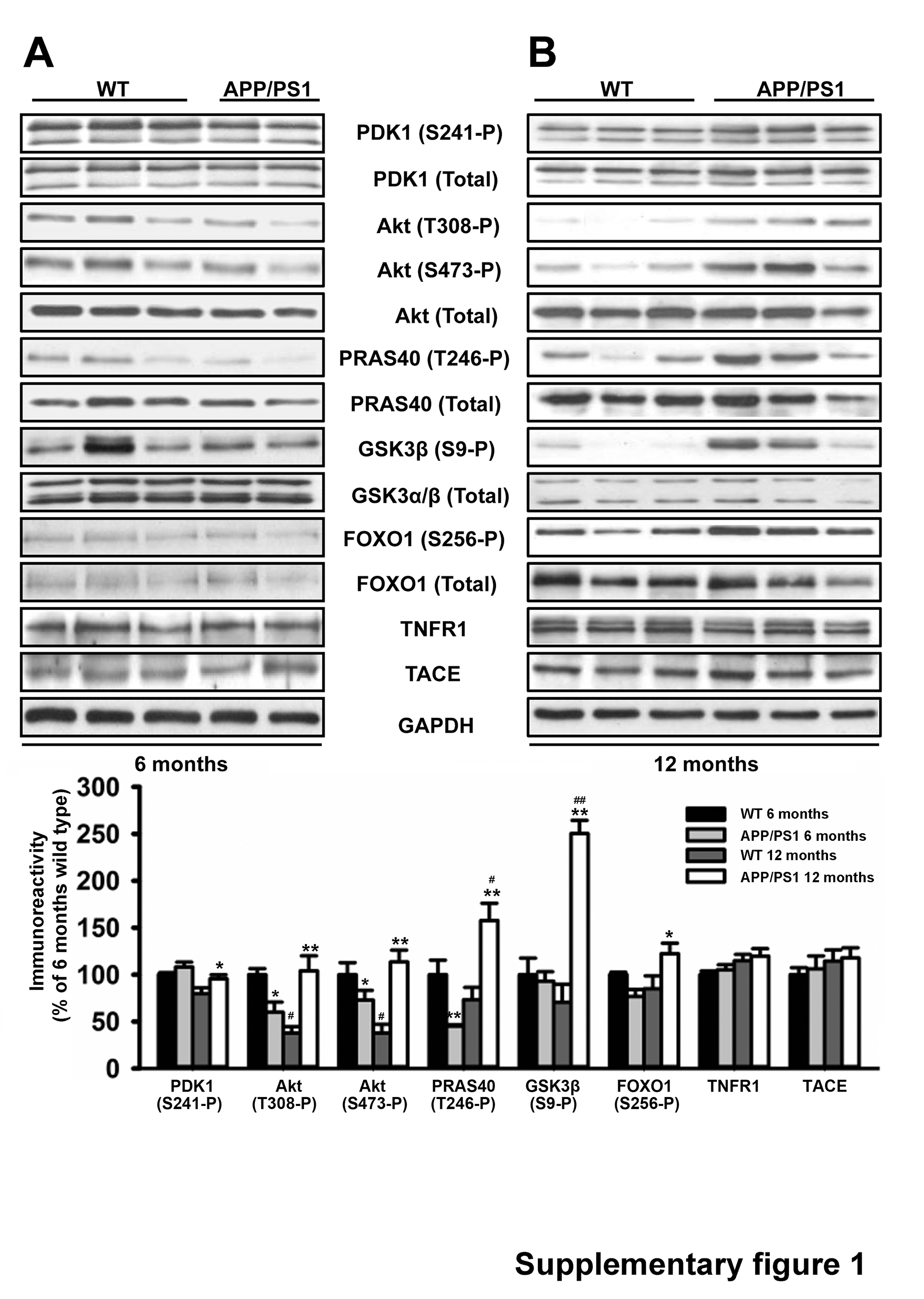

Supplement: Supplementary file 2 [file Image1.TIF]

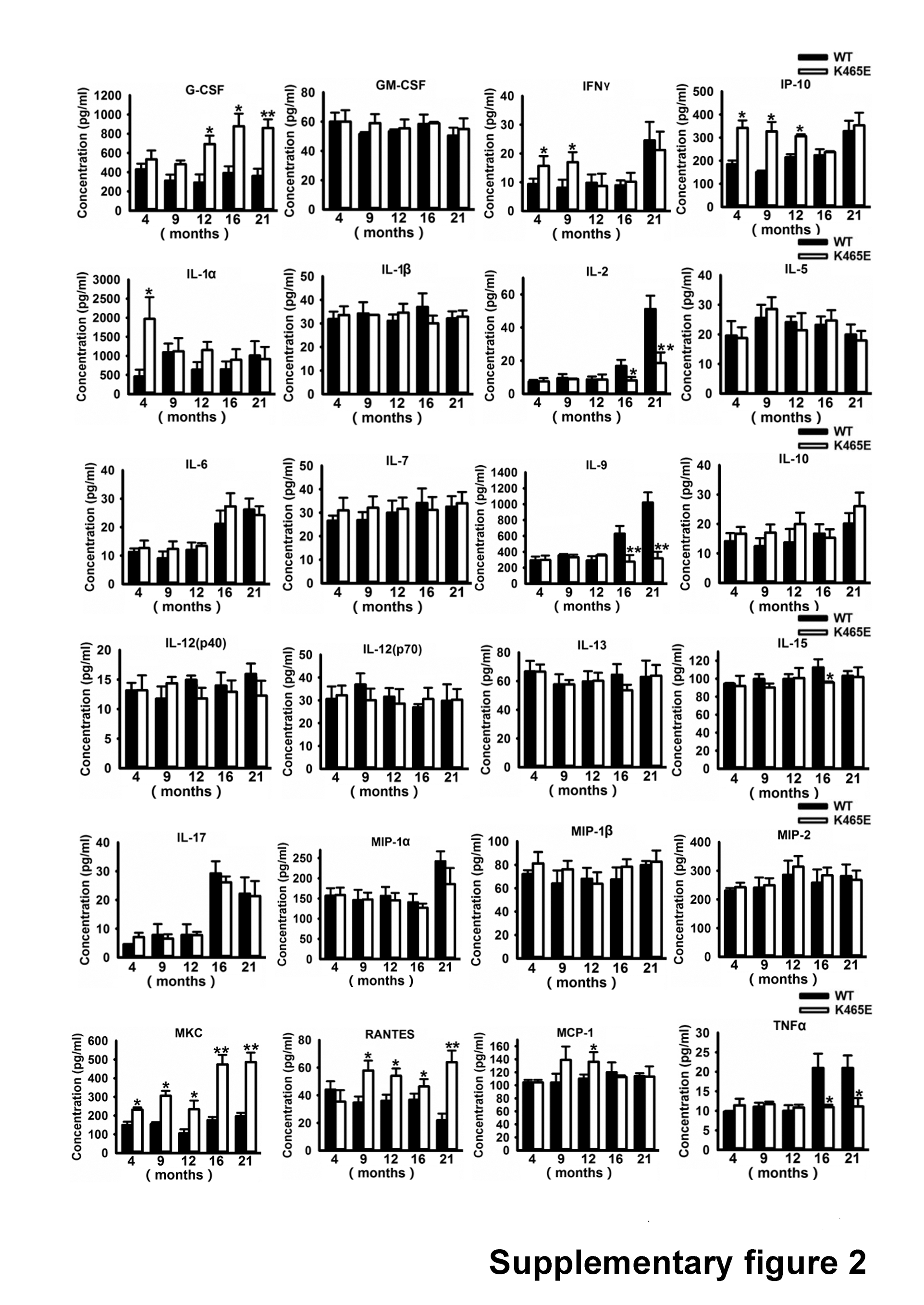

Supplement: Supplementary file 3 [file Image2.TIF]
